# High Dietary Plant Protein Impairs Astaxanthin Pigmentation in Rainbow Trout by Disrupting Cholesterol–Bile Acid Metabolism and Gut Microbiota

**DOI:** 10.3390/ijms262412072

**Published:** 2025-12-15

**Authors:** Alejandro Villasante, Karina Godoy, Elías Figueroa, Héctor Rodríguez, Carolina Ramírez, Paola Orellana, Alberto Sáez-Arteaga, Johana López-Polo, Rafael Opazo, Patricio Dantagnan, Jaime Romero

**Affiliations:** 1 Laboratorio de Biotecnología de Alimentos, Unidad de Alimentos, Instituto de Nutrición y Tecnología de Los Alimentos (INTA), Universidad de Chile, Santiago 7830490, Chile; alejandro.villasante@gmail.com (A.V.);; 2 Facultad de Medicina Veterinaria y Agronomía, Universidad de Las Américas, Santiago 7500000, Chile; 3 Núcleo Científico y Tecnológico en Biorecursos (BIOREN), Universidad de La Frontera, Temuco 4780000, Chile; 4 Núcleo de Investigación en Producción Alimentaria, Departamento de Ciencias Agropecuarias y Acuícolas, Facultad de Recursos Naturales, Universidad Católica de Temuco, Temuco 4781312, Chile; 5 Programa de Anatomía y Biología Del Desarrollo, Facultad de Medicina, Universidad de Chile, Santiago 8380000, Chile; 6 Laboratorio de Nutrición y Fisiología de Peces, Departamento de Ciencias Agropecuarias y Acuícolas, Facultad de Recursos Naturales, Universidad Católica de Temuco, Temuco 4781312, Chile; 7 Facultad de Medicina Veterinaria y Agronomía, Universidad de Las Américas, Concepción 4030000, Chile

**Keywords:** astaxanthin, salmonids, microbiota, cholesterol, bile acids

## Abstract

The replacement of fishmeal with plant-based proteins in aquafeeds is key for sustainable aquaculture but may compromise filet pigmentation in rainbow trout (*Oncorhynchus mykiss*), a quality trait dependent on astaxanthin (Ax) deposition and metabolism. This study aimed to assess the effects of graded fishmeal replacement with a plant protein blend on Ax retention, pigmentation, lipid metabolism, and gut microbiota composition. Rainbow trout were fed three isoenergetic diets containing 60%, 36%, or 12% fishmeal, each supplemented with equal amounts of natural Ax from *Haematococcus pluvialis*, for 12 weeks. Ax retention, pigmentation, plasma metabolites, lipid digestibility, and distal intestinal microbiota were evaluated. The high plant protein diet (12% fishmeal) significantly reduced Ax concentrations in filet and plasma and decreased dorsal and belly pigmentation scores (*p* < 0.05). It also lowered plasma cholesterol and bile acid levels by 18–30%, reduced di-esterified Ax digestibility by 15%, and lipid absorption efficiency by 12%. The gut microbiota shifted significantly, with a marked reduction in *Bacillaceae*, positively correlated with Ax retention and pigmentation. Fish fed High Plant Meal diets exhibited impaired performance parameters, along with reduced lipid accumulation in the liver. High plant meal compromises Ax bioavailability by altering cholesterol–bile acid metabolism and gut microbiota, impairing Ax absorption. However, moderate inclusion of plant meal preserved pigmentation, underscoring the need for dietary cholesterol management and microbiota modulation in plant-based aquafeeds.

## 1. Introduction

In salmonid species, the characteristic pink-red flesh color is among the most relevant criterion of quality [1]. This trait results from the deposition of carotenoid pigments, including astaxanthin (Ax) and canthaxanthin (Cx), in fish skeletal muscle [2]. Carotenoids influence a wide range of functions related to metabolism, antioxidant potential, immune system, reproduction, and growth in fish [3,4]. Wild salmonids obtain carotenoids solely from natural diet (i.e., crustaceans or lower trophic level fish) due to the lack of the enzymatic pathways required for de novo carotenoids synthesis [2]. Hence, carotenoid pigments, predominantly Ax, must be provided through commercial diets, which constitute the single most expensive component, accounting for approximately 6–10% of the total production cost in salmon and pigmented trout farming [1,5]. Commercially available Ax can be obtained either from chemical synthesis (synthetic Ax) or produced naturally by unicellular organisms (e.g., the red yeast *Phaffia rhodozyma*, the bacterium *Paracoccus carotinifaciens*, and the microalga *Haematococcus pluvialis*) [6]. The primary distinction between synthetic-Ax and natural-Ax lies in their chemical structure [7]. Synthetic-Ax is characterized by a non-esterified molecule, commonly referred to as free astaxanthin. In contrast, natural-Ax features a mono or di-esterified molecule, with one or two fatty acids attached to the end of the molecule. These differences in chemical structure imply that natural-Ax is less susceptible to degradation and possesses greater ability to scavenge singlet oxygen and free radicals compared to synthetic-Ax [7]. Although, natural astaxanthin has been suggested to be more stable and to exhibit higher antioxidant activity [8], in the salmon and pigmented trout farming industry, synthetic-Ax is usually preferred because it is both more cost-effective to produce and more efficiently utilized than esterified Ax, since the hydrolysis of ester bonds may constitute an additional step during intestinal digestion/absorption [9,10].

In line with the development of a more sustainable aquaculture industry worldwide, there has been a growing trend in the aquaculture market towards the use of natural-Ax, mostly driven by the rise in demand and consumer’s willingness to pay a premium for natural, high-quality products in the aquaculture market [11,12,13,14]. Dietary supplementation of Ax has been shown to increase hepatic and plasma activities of superoxide dismutase (SOD), catalase (CAT), and glutathione peroxidase (GPx), while reducing lipid peroxidation markers such as malondialdehyde (MDA) [15]. In salmonids, these antioxidant properties of Ax are widely documented and consistently reported across multiple studies [16]. In addition, higher Ax intake has been linked to improved immune competence, with elevated lysozyme activity and complement system responses [12]. Together, these effects underline the dual role of Ax as both a pigmentation agent and a modulator of oxidative stress and immune protection in salmon farming [12,15,16,17].

Previous studies evaluating the utilization of natural-Ax derived from the microalga *Haematococcus pluvialis* have focused on several variables, including absorption [18], digestibility [19], flesh retention and pigmentation [7,19,20,21,22,23,24,25,26,27,28], metabolism [29], growth performance [7,23,28], and health [7,28,29,30] in salmonids. However, relatively little attention has been given to factors such as the use of alternative ingredients to forage fish, particularly terrestrial plant-based proteins, which may affect the bio-utilization efficiency of natural-Ax in salmonid species. It has been suggested that plant-derived meals may decrease lipid digestibility due to the presence of antinutritional factors [31] and/or the disruption of cholesterol–bile acid homeostasis in fish [32,33,34]. Bile acids are endogenous compounds that play relevant roles in animal physiology by enabling biliary secretion of lipids, metabolites, and xenobiotics, as well as facilitating the absorption of fat and liposoluble nutrients in the intestine [35]. Several genes are essential for regulating bile acid homeostasis, which is maintained through a balance of bile acid synthesis, excretion into the gallbladder, intestinal reabsorption, and the return of bile acids to the hepatic sinusoid. In the liver, bile acid production is controlled by the enzymes cholesterol 7α-hydroxylase and cholesterol 7α-hydroxylase, which are encoded by the *cyp7a1* gene and the *cyp8b1* gene, respectively. This process is modulated by the *shp* gene (small heterodimer partner), which suppresses the expression of both *cyp7a1* and *cyp8b1*, thereby inhibiting bile acid synthesis [35,36]. Bile acid excretion into the gallbladder is carried out via the bile salt export pump, which is encoded by the *bsep* gene. In the intestine, through the action of the apical sodium-dependent bile salt transporter and the heteromeric organic solute transporters, encoded by the *asbt* gene and both the *ostα* and *ostβ* genes, respectively, bile acids are transported back to the hepatic sinusoid. Once there, their uptake is predominantly mediated by the Na+-taurocholate co-transporting peptide, which is encoded by the *ntcp* gene [35]. Plant meals may disrupt bile acid homeostasis via two potential mechanisms: first, by causing an unbalanced between excretion/reabsorption of bile acids, and second, via decreasing bile acid synthesis (reducing cholesterol input) [32,33,34]. Indeed, previous work reported that bile acid homeostasis was negatively affected in rainbow trout fed on a soybean meal-based diet, where reduced bile re-absorption rate in hindgut and downregulation in the synthesis of bile acids in the liver of fish was detected [37]. In salmonid species, ingested carotenoids, including Ax, are mostly absorbed in the midgut and hindgut region [5], and their intestinal absorption mechanism remains somehow elusive in fish; however, they are considered similar to mammals, including simple and passive diffusion or endothelial protein transporters [5,38]. Therefore, intestinal absorption of liposoluble pigments, particularly esterified-Ax, may be further reduced in salmonid species by factors affecting lipid digestibility, including feeding fish a High Plant Meal-based diet. In line with this statement, Doughty et al. [23] reported that Chinook salmon (*Oncorhynchus tshawytscha*) fed a mixture of proteins, including wheat meal, corn gluten meal, and poultry meal, in lieu of fishmeal, showed significantly lower redness of the flesh compared with fish fed a fishmeal-based diet supplemented with Ax from the algae *Haematococcus pluvialis*. However, the study did not evaluate the impact of using a plant meal blend as the sole protein source to replace fishmeal on tissue pigmentation in salmon.

Gut microbiota is another factor that has previously been reported to be associated with flesh pigmentation in salmonids [39,40,41]. These studies observed taxa of the gut microbiota, including carotenoid-synthesizing bacterial taxa, to be associated with the red flesh color in salmonids fed a commercial diet. Nguyen et al. [39] identified bacterial taxa at the family level, such as *Bacillaceae*, *Mycoplasmataceae*, *Pseudomonas*, *Phyllobacteriaceae*, and *Comamonadaceae*, as being positively associated with flesh coloration in *Salmo salar*. Ahmed et al. [41] reported that bacterial genera, such as *Leuconostoc lactis*, *Corynebacterium variabile*, *Jeotgalicoccus halotolerans*, and *Leucobacter chromiireducens*, were significantly more abundant in the fecal microbiota of rainbow trout with more pigmented (red) filets. In mammalian models, it was reported that gut microbiota exerted a relevant function in the digestion and absorption of natural-Ax in animals, where antibiotic-induced germ-free animals showed a reduced intestinal absorption rate of natural-Ax [42]. Since it is well-known that gut microbiota diversity and richness is affected when increasing the inclusion levels of plant meals in lieu of fishmeal in salmonids diets [43,44,45,46,47,48], it is worth exploring whether feeding a plant meal-based diet induces changes in gut bacterial taxa composition that might be associated with lower efficiency in using natural-Ax in salmonid species.

Therefore, a study was conducted to test the hypothesis that high fishmeal replacement impairs astaxanthin absorption through concurrent disruptions in lipid digestion, bile acid metabolism, and gut microbiota. While the phenomenon of reduced pigmentation in fish fed high plant-based diets is well documented, the underlying physiological mechanisms—particularly those involving the gut–liver axis, including cholesterol metabolism, bile acid homeostasis, and microbial composition—remain poorly elucidated. This study aimed to address these knowledge gaps. Specifically, we evaluated whether increasing levels of a plant protein blend in the diet of rainbow trout negatively affect the digestibility of mono- and di-esterified Ax derived from a natural source (*Haematococcus pluvialis*), as well as the pigmentation score and Ax concentration in fish flesh. The study also analyzed longitudinal changes in the gut microbiota in response to increasing levels of plant protein inclusion and explored significant correlations between distal gut microbiota taxa and the aforementioned physiological variables at the end of the trial. In addition, hepatic histology and the expression of genes involved in cholesterol and bile acid metabolism in both the liver and hindgut were assessed. Rainbow trout was used as a salmonid model due to its relatively higher efficiency in utilizing dietary carotenoids compared to other salmonid species [2].

## 2. Results

### 2.1. Growth Performance, Productive Parameters, and Chemical Composition of the Whole Body of Fish

**Figure 1:**
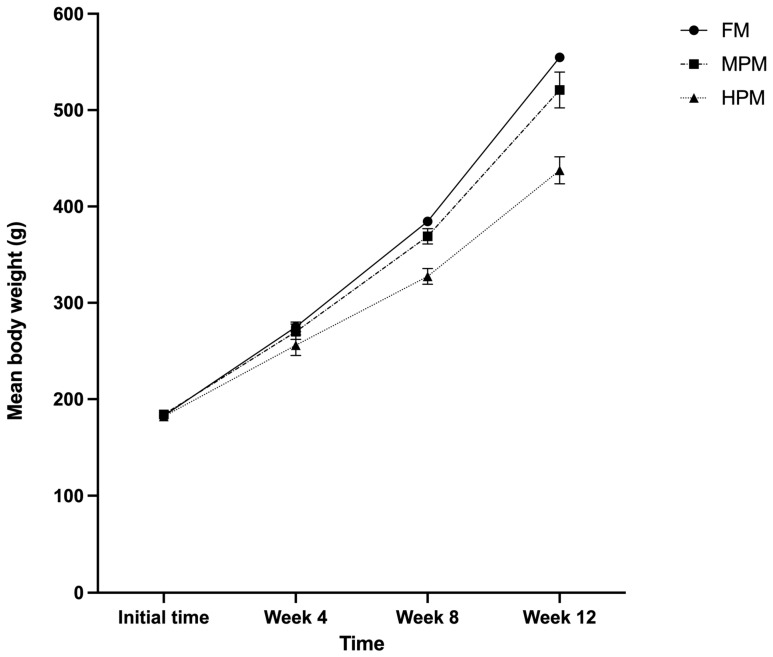
Growth trajectories of rainbow trout monitored over a 12-week feeding trial with three experimental diets: Fish Meal diet (FM), Medium Plant Meal diet (MPM), and High Plant Meal diet (HPM).

Rainbow trout fed either experimental diet showed comparable growth trajectories for the first 4 weeks, but the growth rate strongly diminished for the group consuming the HPM diet after this point (Figure 1). The growth parameters of fish fed the experimental diets are presented in Table 1. Fish fed the HPM diet achieved a significantly lower final body weight compared with fish fed either the FM diet or the MPM diet (*p* = 0.002 and *p* = 0.011, respectively) at the end of the feeding trial. Similarly, weight gain (%/fish) and specific growth rate (SGR) were significantly lower in fish fed either the HPM diet compared with the group fed the FM diet (*p* = 0.003 and *p* = 0.003, respectively) or the MPM diet (*p* = 0.022 and *p* = 0.017, respectively). Conversely, the feed conversion ratio (FCR) was significantly higher in both fish fed the HPM diet and those fed the MPM diet compared with the group fed the FM diet (*p* = 0.028 and *p* = 0.034, respectively). Regarding nutrient retention efficiency, both protein and lipid retention were significantly (*p* = 0.004 and *p* = 0.018, respectively) greater in fish fed the FM diet compared with the group fed the HPM diet. However, when comparing the group fed the MPM diet versus the HPM diet, only lipid retention was significantly greater in the former (*p* = 0.038). For its part, the protein efficiency ratio (PER) was significantly higher in fish fed the FM diet compared with both the group fed the MPM diet and the group fed the HPM diet (*p* = 0.026 and *p* = 0.002, respectively). However, no difference was detected between the latter two groups. The hepatosomatic index (HIS) was significantly (*p* = 0.015) greater in fish fed the FM diet compared with the group fed the HPM diet at the end of the trial. Further, no differences were detected between the group fed the FM diet and the MPM diet or between the group fed the MPM diet and the group fed the HPM diet. The chemical compositions of the whole body of fish fed the experimental diets are summarized in Table 2. The dry matter was significantly higher in fish fed the FM diet compared with both the group fed the MPM diet and the group fed the HPM diet (*p* = 0.015 and *p* = 0.029, respectively). The crude protein content was significantly (*p* = 0.003) greater in fish fed the HPM diet compared with the group fed the FM diet. However, no differences were detected between the group fed the FM diet and the group fed the MPM diet or between the group fed the MPM diet and the group fed the HPM diet. Conversely, the crude fat content was significantly higher in fish fed either the FM diet or the MPM diet compared with the group fed the HPM diet (*p* = 0.004 and *p* = 0.009, respectively). Finally, no differences in ash content were detected between the experimental groups.

### 2.2. Astaxanthin in Pigment Sources, Diet, and Fish Samples (Feces, Muscle, and Plasma)

**Figure 2:**
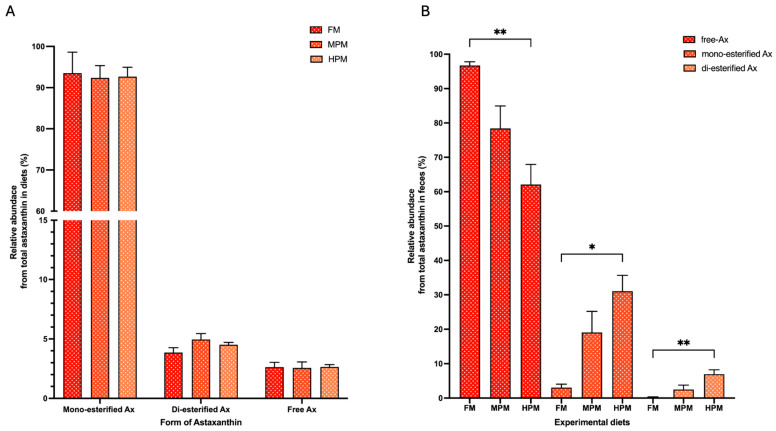
Relative abundance (%) of astaxanthin forms in (**A**) experimental diets and (**B**) feces. Experimental diets include the Fish Meal diet (FM), Medium Plant Meal diet (MPM), and High Plant Meal diet (HPM). * and ** indicate significant differences among diets at *p* < 0.05 and *p* < 0.01, respectively.

**Figure 3:**
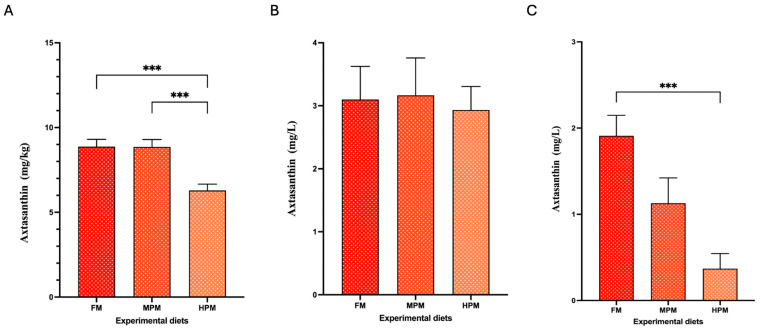
Astaxanthin concentrations in fish fed experimental diets, measured in (**A**) muscle, (**B**) plasma at 24 h postprandial at the beginning of the trial, and (**C**) plasma at 24 h postprandial at the end of the trial. Experimental diets included the Fish Meal diet (FM), Medium Plant Meal diet (MPM), and High Plant Meal diet (HPM). *** indicates significant differences among diets at *p* < 0.001.

The Ax concentrations in the pigment source and experimental diets are summarized in Table 3. The measured concentrations of total Ax were higher than the expected values in the pigment source (130.7 mg/kg vs. 100 mg/kg, respectively). For its part, the values of total Ax in experimental feed were lower than the expected values (ranging from 57 to 62 mg/kg vs. 80 mg/kg, respectively). In the pigment source, the mono-esterified form represented the majority (87%) of the Ax measured, followed by the di-esterified and free forms (10% and 3%, respectively). This agrees with the levels reported by the company for this product (≥95% as Ax esters and ≤5% as free Ax). The experimental diets exhibited a similar trend, with the mono-esterified form accounting for over 92% of total Ax, followed by the di-esterified form at 4–5% and the free Ax at approximately 2–3% (Figure 2A). Conversely, regardless of the experimental diet, free Ax was the predominant form in the feces of fish, accounting for 96.7% in FM, 78.4% in MPM, and 62.1% in HPM. This was followed by the mono- and di-esterified forms, which represented 0.2% and 2.5% in FM and MPM and 6.9% in HPM, respectively. Furthermore, when comparing Ax proportions in feces across experimental groups, the free Ax was significantly (*p* = 0.007) higher in fish fed the FM diet compared to those fed the HPM diet. In contrast, the mono- and di-esterified Ax forms were observed at higher levels in the HPM group compared to the FM diet (*p* = 0.011 and *p* = 0.009, respectively) (Figure 2B). At the start of the trial, Ax concentrations in fish muscle were undetectable. By the end of the trial, however, Ax levels were significantly higher in the muscle of fish fed either the FM diet or the MPM diet compared to those fed the HPM diet (8.9 ± 0.4 m/kg, 8.8 ± 0.4 mg/kg, and 6.3 ± 0.4 mg/kg, respectively; *p* = 0.001 for both comparisons) (Figure 3A). No differences between the group fed the FM diet and the group fed the MPM diet was observed. For its part, at the start of the trial, the Ax concentrations in plasma at 24-postpradial were no different across experimental groups (FM: 3.1 ± 0.5 mg/L, MPM: 3.2 ± 0.6 mg/L, and HPM: 2.9 ± 0.4 mg/L; Figure 3B). However, at the end of the study, the Ax plasma concentration at 24 h postprandially was significantly higher (*p* = 0.001) in fish fed the FM diet compared to those fed the HPM diet at the end of the trial. No significant differences were observed between fish fed the FM diet and those fed the MPM diet nor between fish fed the MPM diet and the HPM diet (FM: 1.9 ± 0.2 mg/L, MPM: 1.1 ± 0.3 mg/L, and HPM: 0.4 ± 0.1 mg/L; Figure 3C).

### 2.3. Apparent Digestibility Coefficient (ADC) of Lipids and Astaxanthin Esters, Muscle Retention of Astaxanthin, and Filet Color from Fish Fed Experimental Diets

**Figure 4:**
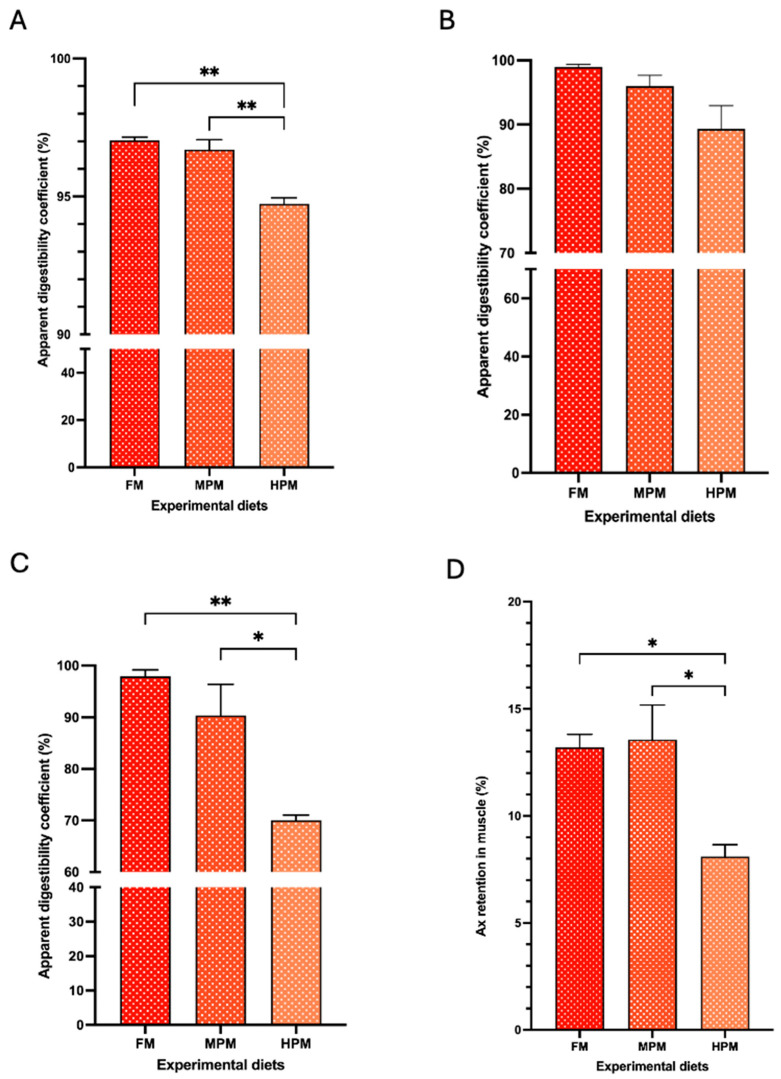
Apparent digestibility coefficient of (**A**) lipids; (**B**) mono-esterified astaxanthin; (**C**) di-esterified astaxanthin; and (**D**) muscle retention of astaxanthin in fish fed experimental diets: Fish Meal diet (FM), Medium Plant Meal diet (MPM), and High Plant Meal diet (HPM). * and ** indicate significant differences among diets at *p* < 0.05 and *p* < 0.01, respectively.

**Figure 5:**
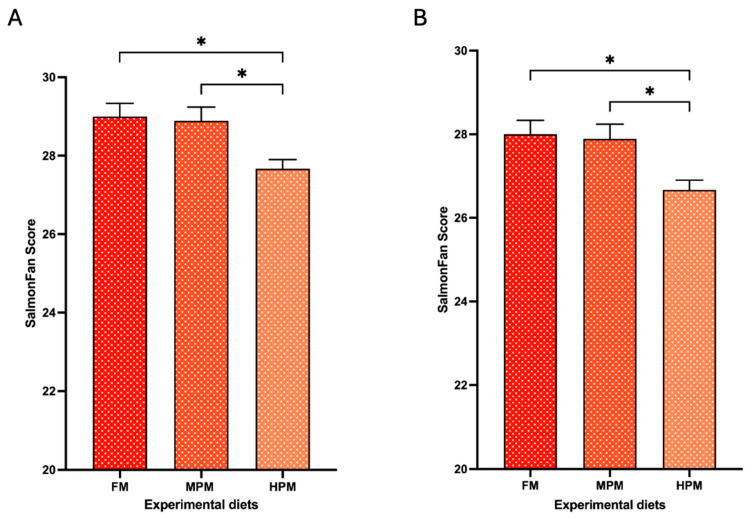
Filet color analysis using the SalmoFan™ score in (**A**) dorsal loin segment and (**B**) belly segment of fish fed experimental diets: Fish Meal diet (FM), Medium Plant Meal diet (MPM), and High Plant Meal diet (HPM). * indicates significant differences among diets at *p* < 0.05.

The apparent digestibility of lipids was significantly higher in fish fed the FM diet (97 ± 0.1%; *p* = 0.002) and the MPM diet (96 ± 0.4%; *p* = 0.004) compared to those fed the HPM diet (94.7 ± 0.2%) (Figure 4A). No significant differences in the apparent digestibility of mono-esterified Ax were observed among the experimental groups (FM: 98.9 ± 0.4, MPM: 95.9 ± 1.7, and HPM: 89.3 ± 3.4). However, a trend (*p* = 0.057) indicating higher digestibility in fish fed the FM diet compared to those fed the HPM diet was noted (Figure 4B). For di-esterified Ax apparent digestibility coefficient, fish fed the FM diet (97.9 ± 1.1%) or the MPM diet (90.4 ± 6.0%) exhibited significantly (*p* = 0.004 and *p* = 0.017, respectively) higher values compared to those fed the HPM diet (70.0 ± 1.0%) (Figure 4C). At the end of the feeding trial, the muscle retention of astaxanthin was significantly higher in fish fed the FM diet (13.2 ± 0.6%) and the MPM diet (13.6 ± 1.6%) compared to those fed the HPM diet (8.1 ± 0.6%) (*p* = 0.03 for both comparisons; Figure 4D). Finally, filet color analysis using the SalmoFan™ score revealed significantly higher pigmentation in both the dorsal loin and belly segments of filets from fish fed either the FM diet (29 ± 0.3; *p* = 0.015 and 28 ± 0.3; and *p* = 0.015, respectively) or the MPM diet (28.8 ± 0.4; *p* = 0.027 and 27.8 ± 0.4; and *p* = 0.027, respectively) compared to those fed the HPM diet (27.6 ± 0.3 and 26.6 ± 0.3, respectively; Figure 5 and Figure S2).

### 2.4. Total Cholesterol and Total Bile Acids in Plasma from Fish Fed Experimental Diets

**Figure 6:**
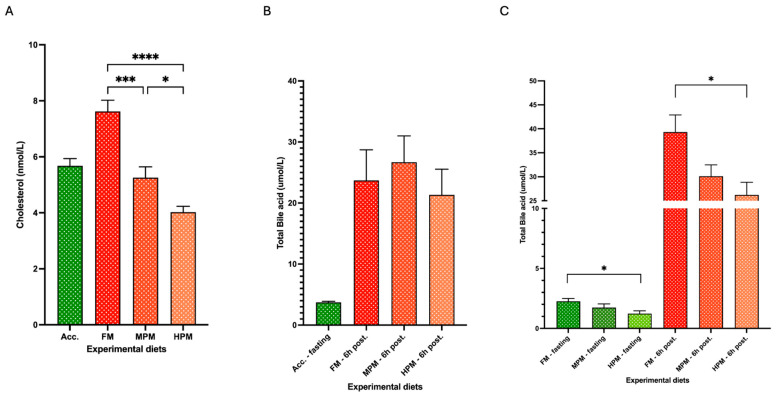
(**A**) Total cholesterol levels under 24 h fasting conditions in fish fed the acclimation diet (Acc.) and the experimental diets at the end of the trial. (**B**) Total bile acid plasma levels under 24 h fasting conditions in fish fed the acclimation diet (Acc.) or 6 h postprandially in fish fed the experimental diets at the beginning of the feeding trial. (**C**) Total bile acid plasma levels under 24 h fasting conditions or 6 h postprandially in fish fed the experimental diets at the end of the feeding trial. Experimental diets: Fish Meal diet (FM), Medium Plant Meal diet (MPM), and High Plant Meal diet (HPM). *, ***, and **** indicate significant differences among diets at *p* < 0.05, *p* < 0.001, and *p* < 0.0001, respectively.

At the end of the study, plasma total cholesterol levels were significantly higher in fish fed the FM diet (7.6 ± 0.4 nmol/L) compared to those fed the MPM diet (5.3 ± 0.4 nmol/L; *p* = 0.0002) or the HPM diet (4.0 ± 0.4 nmol/L; *p* < 0.0001). Additionally, fish fed the MPM diet exhibited significantly higher plasma cholesterol levels compared to those fed the HPM diet (*p* = 0.045; Figure 6A). At the beginning of the trial, no significant differences in total bile acid plasma levels 6 h postprandially were observed among the experimental groups (FM: 23.7 ± 5 nmol/L; MPM: 26.7 ± 4.3 nmol/L; and HPM: 21.3 ± 4.2 nmol/L; Figure 6B). At the end of the feeding trial, during the 24 h fasting state, significantly higher total bile acid plasma levels (*p* = 0.03) were measured in the group fed the FM diet (2.6 ± 0.2 nmol/L) compared to the group fed the HPM diet (1.2 ± 0.2 nmol/L) (Figure 6C). However, no significant differences were detected in 24 h fasting total bile acid plasma levels between fish fed the MPM diet (1.7 ± 0.3 nmol/L) and those fed either the FM diet or the HPM diet. Similarly, in the 6 h postprandial state, significantly higher total bile acid plasma levels (*p* = 0.04) were measured in the group fed the FM diet (39.3 ± 3.4 nmol/L) compared to those fed the HPM diet (26.2 ± 2.6 nmol/L) (Figure 6C). Finally, no significant differences were observed in 6 h postprandial total bile acid plasma levels between fish fed the MPM diet (30.1 ± 2.4 nmol/L) and those fed either the FM diet or the HPM diet.

### 2.5. Gene Expression in Liver and Distal Intestine from Fish Fed Experimental Diets

**Figure 7:**
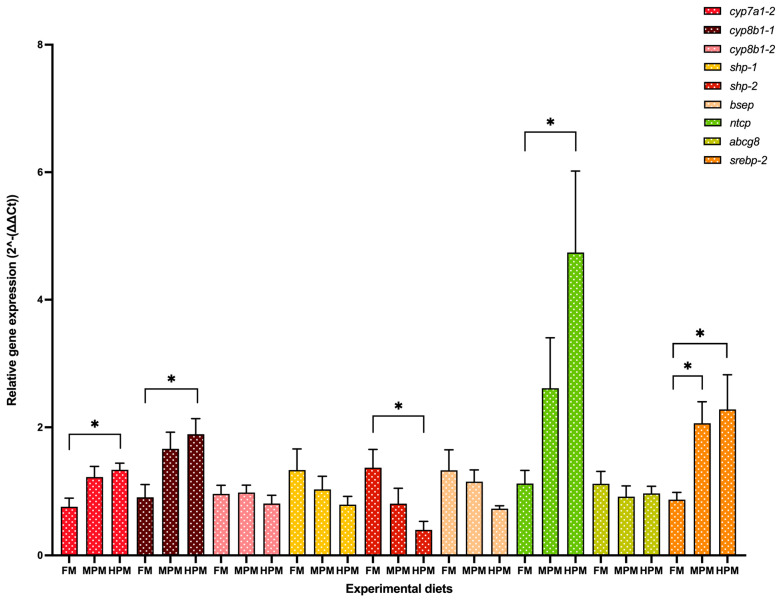
Gene expression analysis measured at 6 h postprandial. Genes of interest analyzed at the end of the 12-week trial in the liver of rainbow trout fed either experimental diet: Fish Meal diet (FM), Medium Plant Meal diet (MPM), and High Plant Meal diet (HPM). Representation of relative mRNA expression of cholesterol 7α-hydroxylase isoform 2 (*cyp7a1-2*), sterol 12α-hydroxylase isoforms 1 and 2 (*cyp8b1-1/-2*), small heterodimer partner isoforms 1 and 2 (*shp-1-7-2*), bile salt export pump (*bsep*), Na+-taurocholate cotransport peptide (*ntcp*), ATP binding cassette transporter (*abcg8*), and sterol regulatory element binding protein (*srebp-2*). Results were normalized to the geometric mean of elongation factor 1-alpha 1 (elf-1a) and b-actin (b-actin) using the delta–delta Ct method 2^−(ΔΔCt)^. Bars represent the mean ± SEM of the relative mRNA expression (n = 9). * indicates significant differences among diets at *p* < 0.05.

**Figure 8:**
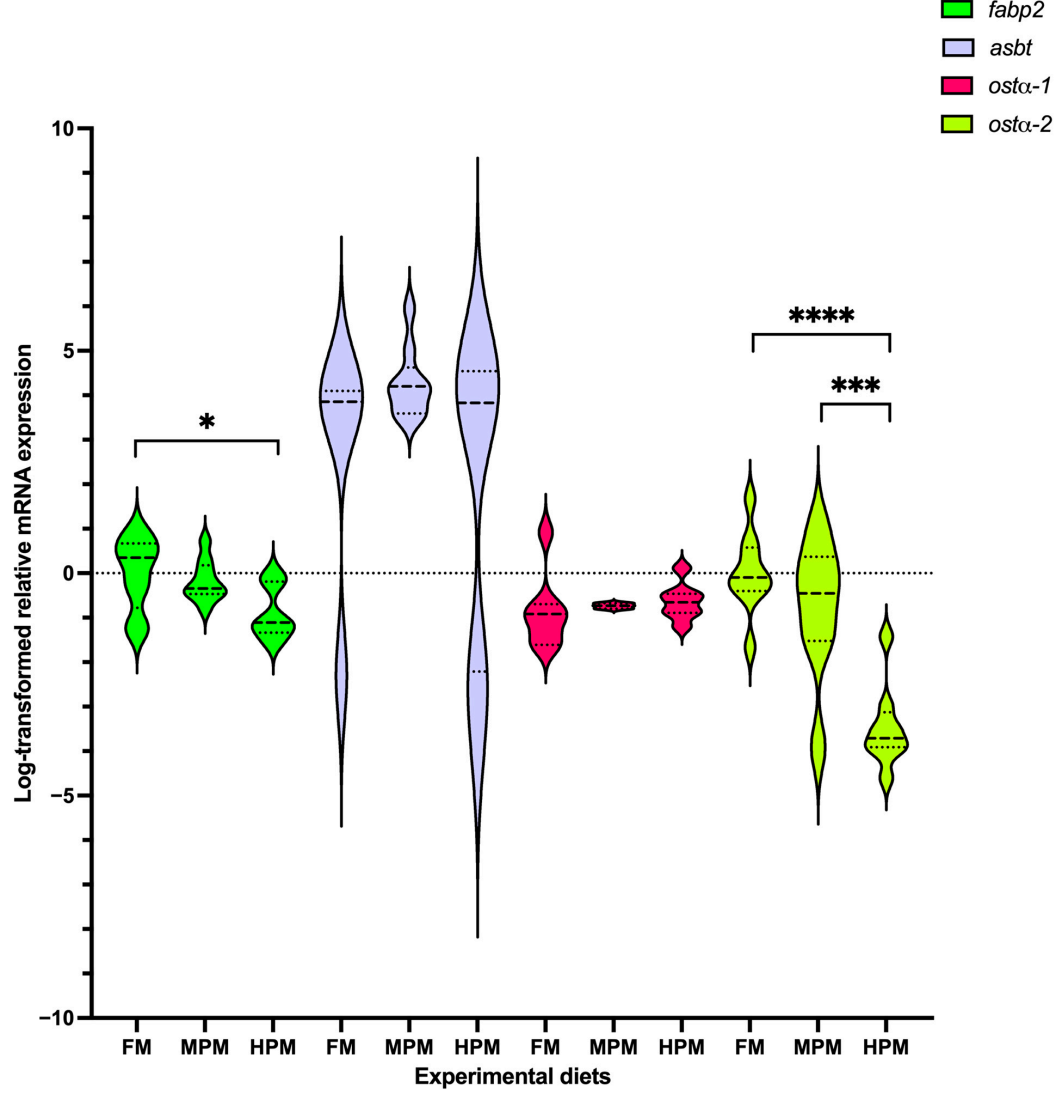
Gene expression analysis measured at 6 h postprandial. Genes of interest analyzed at the end of the 12-week trial in the distal intestine of rainbow trout fed either experimental diet: Fish Meal diet (FM), Medium Plant Meal diet (MPM), and High Plant Meal diet (HPM). Representation of natural log-transformed relative mRNA expression levels of apical sodium-dependent bile salt transporter (*asbt*), heteromeric organic solute transporter isoforms 1 and 2 (*osta -1/-2*), and fatty acid binding protein 2 (*fabp2*). Results were normalized to the geometric mean of elongation factor 1-alpha 1 (elf-1a) and b-actin (b-actin) using the delta–delta Ct method 2^−(ΔΔCt)^. Bars represent the mean ± SEM of the relative mRNA expression (n = 9). The dotted line within each violin represents the median, while the upper and lower dashed lines indicate the first and third quartiles (interquartile range). *, ***, and **** indicate significant differences among diets at *p* < 0.05, *p* < 0.001, and *p* < 0.0001, respectively.

Genes of interest analyzed at the end of the 12-week trial in both the liver and distal of rainbow trout fed either experimental diet are depicted in Figure 7 and Figure 8, respectively. In the liver, both the expression level of the isoforms *cyp7a1-2* and *cyp8b1-1* were significantly (*p* = 0.03 for both comparisons) lower in fish fed the FM diet compared with those fed the HPM diet (Figure 7). However, the expression of both genes was not different between fish fed the MPM diet and those fed either the FM diet or the HPM diet. The expression of the gene encoding for the isoform *cyp8b1-2* showed no differences among experimental groups. A significant upregulation (*p* = 0.016) of the *shp-2* isoform was observed in the group fed the FM diet compared to the fish fed the HPM diet. However, no significant differences were detected when comparing the MPM diet group with either the FM diet group or the HPM diet group. Furthermore, no significant differences were observed in the expression levels of the *shp-1* isoform among any of the experimental groups. The expression levels of the *bsep* gene showed no significant differences between fish fed with either of the experimental diets. The expression level of *ntcp* was significantly lower in fish fed the FM diet compared to those fed the HPM diet. However, no significant differences (*p* = 0.011) were observed between fish fed the MPM diet and those fed either the FM or HPM diets. The expression levels of *abcg8* did not differ significantly between the two experimental groups. Significant upregulation of *srebp-2* was observed in fish fed the HPM and FM diets compared with those fed the MPM diet (*p* = 0.043 and *p* = 0.03, respectively). However, no significant differences were detected in the expression levels of *srebp-2* between fish fed the HPM diet and those fed the MPM diet. In the distal intestine (Figure 8), the expression level of *fasbp-2* was significantly (*p* = 0.019) greater in the group fed the FM diet compared with the group fed the HPM diet; however, no differences were detected between fish fed the MPM diet and those fed the HPM diet. The expression level of the *asbt* gene showed no significant differences among the experimental groups. Finally, the expression level of isoform 1 of the *ostα* gene was significantly downregulated in the group fed the HPM diet compared both with the group fed the FM diet and the group fed the MPM diet (*p* < 0.0001 and *p* = 0.0003, respectively). However, no difference was detected between fish fed the FM diet and those fed the MPM diet. No significant differences were observed in the expression level of isoform 2 of the *ostα* gene across the experimental groups.

### 2.6. Distal Intestine Microbiota of Fish Fed Experimental Diets

#### 2.6.1. High-Throughput Sequence Data

A total of 1169 ASVs were detected in the dataset comprising 109 samples. After removing ASVs not assigned at the phylum level, as well as those belonging to *Chloroflexi* and *Cyanobacteria*, 1068 ASVs were retained. Following the removal of singleton ASVs, 434 ASVs were kept. These ASVs constituted the dataset used for beta diversity and relative abundance analyses. Rarefaction curves indicated that all samples reached a plateau, confirming that the sequencing depth was adequate (Figure S3).

#### 2.6.2. Diversity Analysis of Distal Intestine Digesta Microbiota Between Experimental Groups

**Figure 9:**
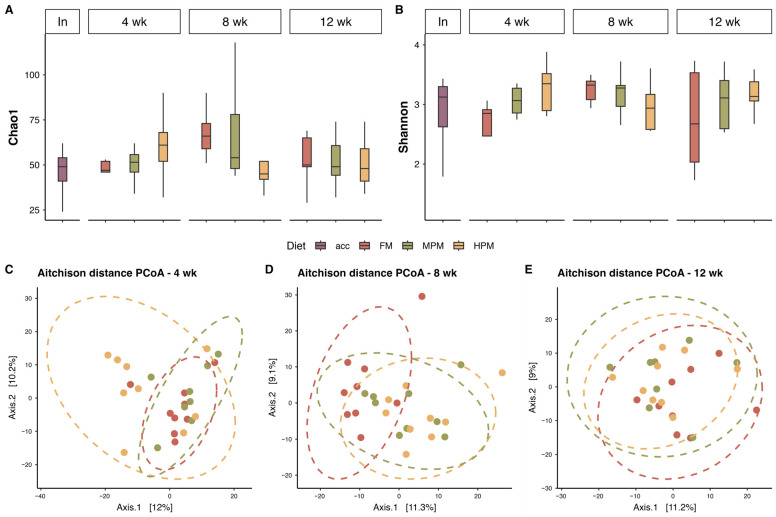
Microbiota diversity in digesta samples over the course of the study. Panels (**A**) and (**B**) represent richness and diversity indices, respectively. Panels (**C**–**E**) show PCoA plots based on Aitchison distance for different sampling weeks. Abbreviations: In, initial time; 4 wk, week 4; 8 wk, week 8; and 12 wk, week 12. Acclimation diet (acc), Fish Meal diet (FM), Medium Plant Meal diet (MPM), and High Plant Meal diet (HPM). In the lower panels (**C**–**E**), each point represents the microbial community composition of an individual fish at a given sampling time, projected onto Principal Coordinates Analysis (PCoA) plots based on Aitchison distance. The dashed ellipses represent 95% confidence intervals around the multivariate centroid of each diet group, while the dotted ellipses illustrate the spread and clustering of samples within each treatment, facilitating visualization of group-level beta-diversity patterns.

Alpha diversity indices showed no significant differences in digesta samples (two-way ANOVA, diet: *p* > 0.05; time: *p* > 0.05; and diet × time: *p* > 0.05) over the course of the feeding trial (Figure 9). Similarly, no significant differences in terms of richness and diversity of the microbiota found in the water samples at the end of the 12-week feeding trial were observed (Figure S4). Significant differences in beta diversity were detected through PERMANOVA analysis in digesta samples among all dietary treatments at week 8 and between the FM diet and the HPM diet at the end of the trial (Table 4). At week 4, Betadisper results revealed significant differences, suggesting that the observed dietary differences at this time point may be attributed to sample dispersion within the groups. For its part, significant differences in beta diversity were detected through PERMANOVA analysis in water samples when comparing the initial fish population fed the acclimation diet with those fed the experimental diet at the end of the trial. However, no differences in microbiota composition were observed in water samples between the experimental groups at the end of the feeding trial (Table S1).

#### 2.6.3. Microbiota Composition

**Figure 10:**
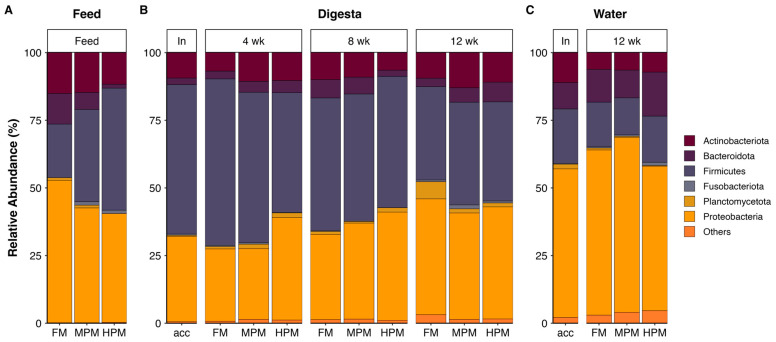
Microbiota composition at the phylum level (**A**) in “Feed” samples; (**B**) in the “Digesta” sample based on administered diets; and (**C**) “Water” samples. Average relative abundance per diet for “Digesta” and “Water” samples (n = 9 and n = 3, respectively). Phyla with a relative abundance of less than 1% were grouped under the category “Others.” Acclimation diet (acc), Fish Meal diet (FM), Medium Plant Meal diet (MPM), and High Plant Meal diet (HPM).

**Figure 11:**
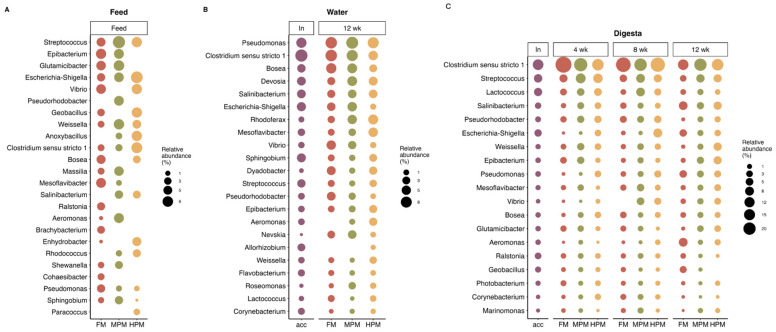
Microbiota composition at the genus level (**A**) in feed samples; (**B**) water samples; and (**C**) digesta samples, respectively, based on administered diets. Average relative abundance at the genus level (for digesta, n = 9 and for water, n = 3, respectively), including genera present in four or more replicates in at least one diet and with a minimum average relative abundance of 2%. Acclimation diet (acc), Fish Meal diet (FM), Medium Plant Meal diet (MPM), and High Plant Meal diet (HPM).

The most represented phyla in the microbiota of digesta, feed, and water samples were *Proteobacteria*, *Firmicutes*, and *Actinobacteria* (Figure 10). At the genus level, feed samples showed differences in the hierarchy of the most abundant genera (Figure 11A). In the FM diet, the genus *Epibacterium* was the most abundant (7.4%), followed by *Glutamicibacter* (6.2%) and *Streptococcus* (5.1%). In the MPM diet, *Streptococcus* was the most abundant genus (11.8%), followed by *Glutamicibacter* (8%) and *Weissella* (7.7%). In the HPM diet, the most abundant genera were *Escherichia-Shigella* (10.7%), *Clostridium sensu stricto 1* (8.8%), and *Geobacillus* (7.5%). Similarly, in the water samples, differences in the hierarchy of the most abundant genera were observed (Figure 11B). At the beginning of the trial (acclimatation diet; acc), the most abundant genera were *Clostridium sensu stricto 1* (10.7%), *Pseudomonas* (6.5%), and *Escherichia-Shigella* (4.7%). At the end of the trial, in water samples from the group fed the FM diet, the most abundant genera were *Clostridium sensu stricto 1* (9.2%), *Pseudomonas* (9%), and *Vibrio* (5.2%). In the group fed the MPM diet, the most abundant genera in the water samples were *Pseudomonas* (10.1%), *Clostridium sensu stricto 1* (8.3%), and *Devosia* (5.9%), whereas in the group fed the HPM diet, the most abundant genera were *Pseudomonas* (8.3%), *Rhodoferax* (6.1%), and *Mesoflavibacter* (5.3%). Finally, differences in the hierarchy of the most abundant genera in the digesta samples were observed when considering the sample time and/or experimental group (Figure 11C). At the beginning of the trial, the most abundant genera were *Clostridium sensu stricto 1* (13.7%), *Streptococcus* (6.9%), and *Lactococcus* (6.4%). In week 4, *Clostridium sensu stricto 1* was the most abundant genus across the experimental groups, with the highest abundance observed in the FM group (39.2%), followed by the MPM group (24.4%) and the HPM group (16.9%). *Streptococcus* and *Lactococcus* were the second and third most abundant genera in the experimental groups. *Streptococcus* was most abundant in the group fed the MPM diet (11%), followed by the HPM group (9.7%) and the FM group (5.9%), whereas *Lactococcus* was more abundant in the HPM (5.3%), followed by the MPM group (4.9%) and the FM group (3.5%). In week 8, *Clostridium sensu stricto 1* was the most abundant genus across the experimental groups, with the highest abundance observed in the FM group (31.6%), followed by the HPM group (29.7%) and the MPM group (21%). However, the second and third most abundant genera varied across the experimental groups. In the FM group, *Streptococcus* (4.6%) and *Pseudorhodobacter* (3.7%) were the second and third most abundant genera, respectively. For its part, the second and third most abundant genera in the MPM group were *Streptococcus* (7.7%) and *Lactococcus* (7.5%), while in the HPM group, they were *Escherichia-Shigella* (8.3%) and *Streptococcus* (5.8%), respectively. Similarly, at the end of the trial (week 12), *Clostridium sensu stricto 1* was the most abundant genus across the experimental groups in digesta samples, with the highest abundance observed in the MPM group (21%), followed by the HPM group (17%) and the FM group (13.3%). However, differences in the second and third most abundant bacterial genera were observed across the experimental groups. In the group fed the FM diet, the second and third most abundant genera were *Aeromonas* (7.8%) and *Salinibacterium* (7.5%), respectively. In the MPM group, they were *Streptococcus* (6.5%) and *Salinibacterium* (4.1%), while in the HPM group, they were *Weissella* (6.3%) and *Salinibacterium* (6.3%).

#### 2.6.4. Correlations Between Microbiota Taxa and Variables of Interest

**Figure 12:**
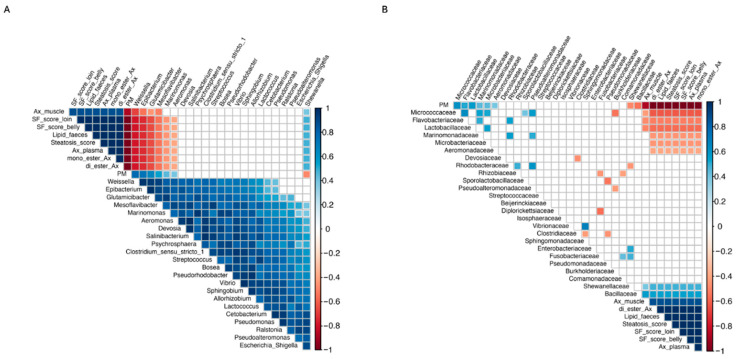
Spearman correlations between microbiota components and additional variables at the final sampling time (week 12) (**A**) at the genus level and (**B**) at the family level. This analysis included genera present in 4 or more samples per diet, in at least 1 diet, and with a relative abundance greater than 1%. Absolute abundance data were transformed using the CLR method prior to performing the correlations. Only significant correlation values are displayed, with red indicating significant negative correlations and blue indicating significant positive correlations.

Spearman correlations between microbiota components and additional variables that were significant (*p* < 0.05) at the final sampling time are depicted at the genus level in Figure 12A and at the family level in Figure 12B, respectively. Correlation *p*-values are provided in the Supplementary Materials (Table S2 for the family level and Table S3 for the genus level). At the bacterial genus level, *Weissella*, *Epibacterium*, *Glutamicibacter*, and *Mesoflavibacter* showed significant negative correlations with Ax concentration in muscle, pigmentation scores in the loin and belly of the filet, Ax concentration in plasma, lipid content in feces, steatosis score, and the digestibility of mono- and di-esterified Ax. Similarly, *Marinomonas* and *Aeromonas* exhibited significant negative correlations with variables including pigmentation scores in the loin and belly of the filet, Ax concentration in plasma, lipid content in feces, steatosis score, and the digestibility of mono- and di-esterified Ax. In contrast, *Shewanella* showed a significant positive correlation with all the previously mentioned variables. Furthermore, *Weissella*, *Epibacterium*, *Glutamicibacter*, *Mesoflavibacter*, *Marinomonas*, and *Aeromonas* showed significant positive correlations with the inclusion level of the plant protein blend in the diet, whereas Shewanella exhibited a significant negative correlation. For its part, at the bacteria family level, *Shewanellaceae* and *Bacillaceae* revealed significant positive correlations with the Ax concentration in muscle, pigmentation scores in the loin and belly of the filet, Ax concentration in plasma, lipid content in feces, steatosis score, and the digestibility of mono- and di-esterified Ax and significant negative correlations with the inclusion level of the plant protein blend in the diet. On the contrary, *Micrococcaceae*, *Flavobacteriaceae*, *Lactobacillaceae*, *Marinomonadaceae*, *Microbacteriaceae*, and *Aeromonadaceae* showed significant negative correlations with the all the productive previously mentioned variables and significant positive correlations with the inclusion level of the plant protein blend in the diet.

#### 2.6.5. Differences in the Digesta Microbiota Composition in Fish Fed Experimental Diets

**Figure 13:**
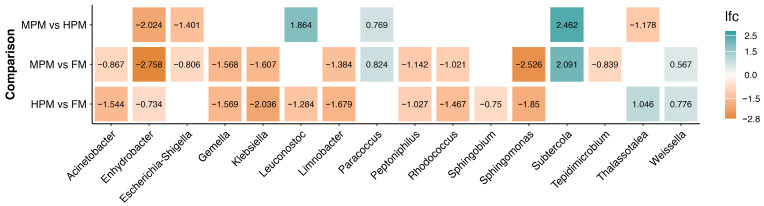
ANCOMBC2 results at the genus level, using the “fix_formula” option (Diet + Time). Log fold change (lfc) values correspond to pairwise comparisons, including only genera with significant differences between diets.

**Figure 14:**
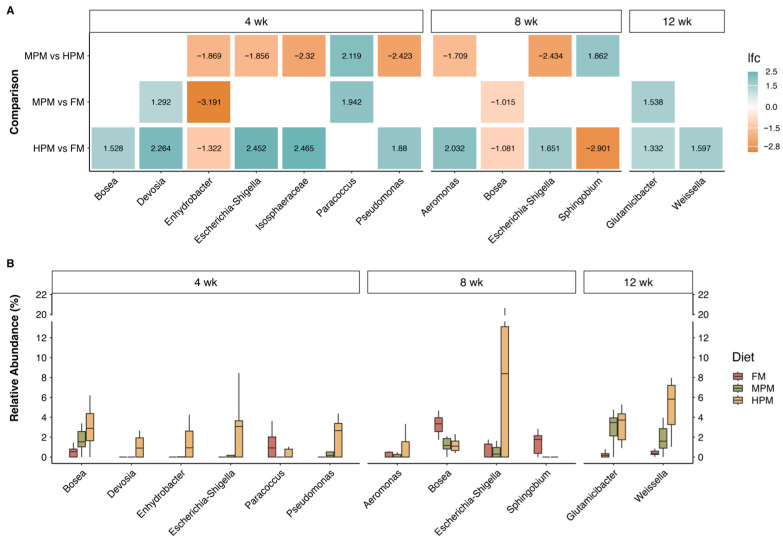
ANCOMBC2 results at the genus level analyzed by sampling week. (**A**) Log fold change (lfc) values correspond to pairwise comparisons, including only genera with significant differences between diets; (**B**) relative abundance of genera with significant differences between diets, as identified by ANCOMBC2. Experimental diets: Fish Meal diet (FM), Medium Plant Meal diet (MPM), and High Plant Meal diet (HPM).

Differential abundance of microbiota components across administered diets was evaluated using ANCOMBC2. Analyses were conducted on two datasets: “digesta” and “water.” For the “digesta” dataset, initial time point samples were excluded, focusing only on samples from weeks 4, 8, and 12. For the “digesta” dataset, two approaches were taken: the first included the covariate time, using the “fix_formula” option (Diet + Time) to perform comparisons based on the diet variable. In the second approach, the “digesta” dataset was split by week, and comparisons between diets were then conducted. The ANCOMBC2 results revealed significant differences in the “digesta” dataset samples between diets across various taxonomic levels. At the genus level, 16 differentially abundant taxa were identified using the first approach (Figure 13). When the dataset was separated by weeks, 10 genera showed significant differences between diets, with the highest number (6) observed during the first sampling week. In contrast, only two genera exhibited differences in week 12 (Figure 14).

### 2.7. Hepatic Histology Evaluation

**Figure 15:**
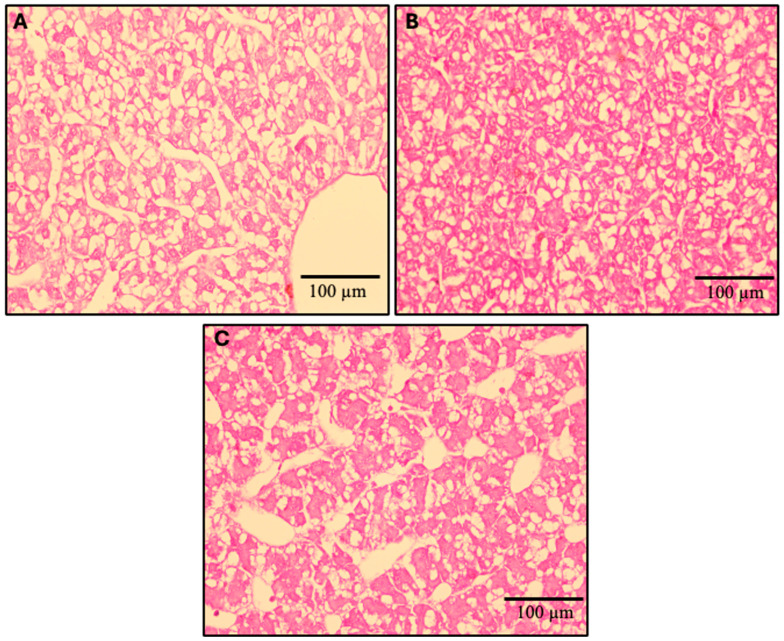
Histological analysis. Liver sections of rainbow trout (n = 9), stained with hematoxylin/eosin, were examined following exposure to experimental diets with increasing dietary inclusion levels of plant protein blend at the end of the feeding trial. (**A**) Liver sections from fish fed the Fish Meal diet (FM) for 12 weeks; (**B**) liver sections from fish fed the Medium Plant Meal diet (MPM) for 12 weeks; and (**C**) liver sections from fish fed the High Plant Meal diet (HPM) for 12 weeks. In (**A**) hepatic tissue with greater fat infiltration, cytoplasmic macrovacuoles, and a noticeable displacement of nuclei towards the cell membrane; (**B**) shows hepatic tissue with moderate fat infiltration, containing cytoplasmic microvacuoles and hepatocytes with centrally located nuclei; and (**C**) shows hepatic tissue with low fat infiltration, and hepatocytes exhibit an initial stage of cytoplasmic microvacuoles.

**Figure 16:**
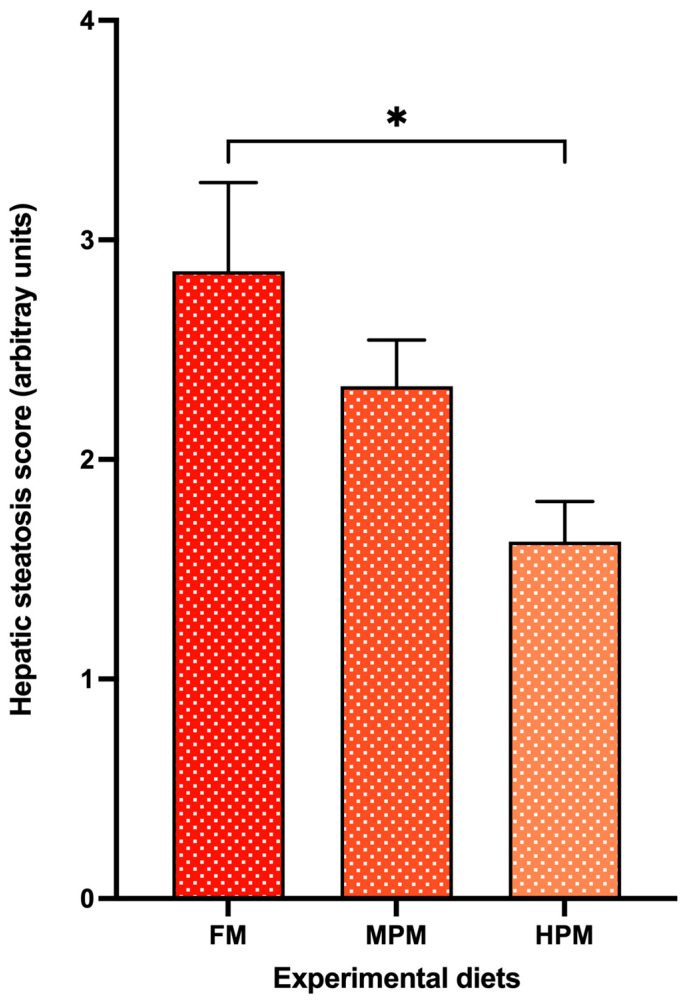
A semi-quantitative scoring system was used to assess fat content in hepatic tissue of rainbow trout fed experimental diets: Fish Meal diet (FM), Medium Plant Meal diet (MPM), and High Plant Meal diet (HPM). Based on visible hepatic lipid droplet-derived vacuoles within fish hepatocytes, the observed median scores for the FM, MPM, and HPM diets were 3, 2, and 2 arbitrary units, respectively. * indicates significant differences among diets at *p* < 0.05.

The hepatic histomorphology of fish fed experimental diets over the 12-week feeding trial is presented in Figure 15. Using the semi-quantitative scoring system to assess fat content based on visible hepatic lipid droplet-derived vacuoles within fish hepatocytes, the observed median scores for the FM, MPM, and HPM diets were 3, 2, and 2, (arbitrary units), respectively (Figure 16). A Kruskal–Wallis rank sum test identified significant differences in liver steatosis scores among the experimental groups (*p* < 0.018). Additionally, pairwise comparisons conducted with the Wilcoxon rank sum test and adjusted using Bonferroni’s correction indicated that the median liver steatosis score in the FM group differed significantly from that in the HPM group (*p* < 0.023). However, no significant difference was observed between the FM group and the MPM group or the MPM group compared with the HPM group.

## 3. Discussion

This study examined how replacing fishmeal with a plant protein blend in rainbow trout diets influences the digesta microbiota and its potential role in astaxanthin metabolism and pigmentation. Our results showed that replacing half of the fishmeal in feeds with a plant protein blend had no impact on Ax concentration in the muscle or flesh pigmentation in trout. However, higher inclusion levels of plant proteins negatively affected both variables in trout (Figure 3A, Figure 5A and Figure 5B, respectively). Consistent with this observation, Doughty et al. [23] found that Chinook salmon (*Oncorhynchus tshawytscha*) fed a low fishmeal diet—partially replaced with wheat meal, corn gluten meal, and poultry meal—exhibited significantly reduced flesh redness compared to those fed a high fishmeal diet. Previous findings also indicated that muscle color and astaxanthin retention were adversely affected by high levels of corn gluten meal despite proper dietary astaxanthin levels [49]. In the present study, both the pigmentation score and Ax concentration in the flesh of fish fed experimental diets were positively associated with the Ax concentration in plasma (Figure 12). These findings are consistent with previous studies that detected a strong correlation between postprandial plasma concentrations of Ax and the retention of Ax in the flesh of salmonid species fed varying dietary levels of this pigment [50,51]. Moreover, Kiessling et al. [52] suggested that the level of Ax in the blood could serve as a suitable predictor of long-term astaxanthin deposition in muscle tissue in Atlantic salmon due to the strong correlation between Ax levels in blood and muscle. Hence, in our study, variations in the bioavailability of Ax might account for the differences observed in its deposition in muscle tissue among the experimental groups, particularly when comparing the control group with fish fed the HPM diet. However, it is important to consider that other works have reported a weak correlation between plasma and muscle carotenoids concentration [53] or between the flesh pigmentation score and Ax concentration in plasma in salmonids species [54]. This discrepancy might be attributed to differences in factors related to experimental designs, including fish species, fish size, water salinity, feed intake, dietary composition, dietary lipid level, and postprandial blood sampling time, among others. Nonetheless, the plasma concentration of carotenoids, including Ax, is considered a reliable indicator of their availability for muscle pigmentation in salmonid fish, as it closely correlates with both dietary carotenoid levels and their deposition in muscle tissue [55].

Previous research has documented reduced lipid digestibility in salmonid species fed diets with high levels of plant proteins, along with minor declines in growth and feed conversion efficiency [1,32,34,56,57]. In the present study, both lipid and di-esterified Ax digestibility were significantly hindered in fish fed the highest inclusion level of the plant protein blend (Figure 4A and Figure 4C, respectively). Although we observed no differences in the ADC of mono-esterified Ax between experimental groups, a strong trend towards lower digestibility in fish fed the HPM diet compared with the control group was detected (Figure 4B). These results indicate that Ax molecules with a higher degree of esterification may be more difficult to hydrolyze than those with lower esterification, especially under conditions of compromised lipid digestibility in the fish intestinal environment, resulting in the reduced effectiveness in pigmenting fish flesh. Since free astaxanthin is the only form detected in the flesh of rainbow trout, astaxanthin esters must undergo prior hydrolysis in the digestive tract to be absorbed in their free form [2]. Hence, the hydrolysis rate of astaxanthin esters, which releases free astaxanthin, appears to be a limiting factor in the efficient retention of astaxanthin in muscle in salmonids fed pigment sources rich in esterified Ax forms [9,58]. Schiedt and Leuenberger [59] and Schiedt et al. [60] described that synthetic astaxanthin di-palmitate was significantly less effective in pigmenting salmonids when compared with the unesterified Ax form. Similarly, Foss et al. [61] reported that rainbow trout and sea trout achieved more efficient pigmentation when fed diets supplemented with free astaxanthin compared to those fed diets containing astaxanthin diesters.

Notably, in this study, free astaxanthin (Ax) was found in higher proportions in the feces, whereas in the experimental diets, it was present in lower proportions. This inversion likely reflects the digestion process, which converts the esterified Ax into its free form. Additionally, we observed an increased proportion of mono- and di-esterified forms of astaxanthin (Ax) in the feces of the group fed the higher plant meal blend compared to the control group (Figure 3A and Figure 3B, respectively). These findings suggest that elevated plant meal levels hinder the hydrolysis of esterified astaxanthin (Ax) molecules from the pigment source, which may account for the decrease in the di-esterified Ax digestibility and the Ax retention efficiency in the group fed the High Plant Meal blend, relative to both the FM and MPD diet groups (Figure 4C and Figure 4D, respectively). Interestingly, the mean ADC values for both esterified forms of Ax (mono- and di-esterified) exceeded 90% in the group fed the fishmeal-based diet, surpassing previously reported values for natural Ax forms and being close to the digestibility values (>95%) reported for free Ax in trout [5,9,19,21,22,53,61]. While caution was taken during sample collection and analysis in our study, factors like carotenoid decomposition during fecal collection, insufficient extraction of carotenoids, and variability in analytical methods must be acknowledged as potential contributors to overestimate digestibility [2]. The above notwithstanding, the discrepancy in ADC values for the esterified Ax forms between our study and previous research may be attributed to differences in the pigment source, analytical techniques, or the dietary inclusion protocol, among others, which have been cited as factors that could account for the inconsistencies across studies [5]. Lower carotenoid retention rates in rainbow trout fed natural esterified Ax from *Haematococcus pluvialis* compared to synthetic astaxanthin have been attributed to the algal cell wall, suggesting that it hindered the efficient absorption of the total carotenoid extract from the microalga. On the contrary, in our study, the pigment source used was a cell wall-free, oil-based extract derived from *Haematococcus pluvialis*, added at the oil incorporation step during feed preparation. This is particularly relevant, as carotenoid bioavailability is closely linked to the food matrix and the incorporation process. In our study, these factors likely enhanced the intestinal bioavailability of the Ax forms, supporting their hydrolysis and absorption at the site of digestion. In line with this claim, Bowen et al. [20] found no differences in the utilization efficiency of isolated *Haematococcus* mono- and diesters of Ax compared to a synthetic free Ax in rainbow trout. Moreover, the authors reported no significant differences in the postprandial (16 and 24 h) mean serum Ax concentration between fish fed the free Ax and fish fed the purified esterified Ax after being fed a single meal.

Increasing evidence suggests that feeding plant-based proteins reduces lipid digestibility by disrupting bile acid homeostasis in carnivorous fish, including salmonid species [33,34]. Plant meal components like non-starch polysaccharides (NSPs) have been described to increase intestinal viscosity and promote bile acid excretion via feces in salmonid species [62,63]. Furthermore, earlier studies have demonstrated that short amino acid residues derived from soybean protein possess a strong bile acid-binding ability [64]. This binding capacity may hinder bile acid reabsorption, potentially leading to increased bile acid loss through feces in teleosts consuming diets rich in soybean meal [37]. In the present study, the 6 h postprandial circulating total bile acid levels at the end of the feeding trial were significantly lower in the group fed the high plant protein diet compared to the FM group (Figure 6C). Notably, this difference was absent at the beginning of the trial when fish were first exposed to a single meal of their assigned experimental diets (Figure 6B). Considering the comparable crude fiber content, its uniformly low levels across the diets, and the significantly reduced fasting plasma total bile acid concentrations in fish consuming the HPM diet relative to those on the FM diet by the end of the trial, the postprandial decrease in plasma total bile acid concentrations associated with higher plant-derived protein inclusion is more likely a result of impaired bile acid biosynthesis rather than the action of feed components facilitating bile acid excretion through feces. Bile acid production is regulated by a negative feedback loop, where elevated bile acid concentrations suppress the expression of *cyp7a1* and *cyp8b1* through activation of the small heterodimer partner (SHP), encoded by the *shp* gene, thereby reducing bile acid synthesis [65]. Our findings show that the negative feedback loop was active in fish fed the FM diet. However, increasing the inclusion of the plant protein blend relieved the suppression of the feedback loop by downregulating the expression of the *shp-2* isoform while upregulating *cyp7a1* and *cyp8b1* expression compared to the FM group (Figure 7). This likely represents a compensatory response to the decreased bile acid concentrations detected in the group receiving the High Plant Meal diet. Interestingly, the exact opposite was observed by Murashita et al. [37], where fish fed a soybean meal-based diet long term exhibited downregulation of the bile acid synthesis-associated genes. However, several factors may have contributed to the differing bile physiology responses to dietary fishmeal replacement with a plant protein blend, including the substantially higher inclusion level of SBM in the other study (53% vs. 18%). This is especially true since soybean meal contains some antinutritional factors, such as soya-saponin, which has been described to suppress the expression of *cyp7a1*, hence decreasing the bile acid synthesis in Atlantic salmon [66]. The level of extruded soybean meal inclusion may not have been high enough to introduce substantial quantities of soya-saponin capable of adversely affecting the transcriptional activity of genes associated with bile acid synthesis in the present study. The transcription of *ntcp*, a Na^+^-taurocholate co-transporting polypeptide that facilitates bile acid uptake by hepatocytes, is suppressed via an indirect mechanism in which bile acids activate the farnesoid X receptor (FXR). The activation of FXR induces the expression of *shp*, which in turn prevents the formation of the heterodimer complex between the retinoic acid receptor and the retinoid X receptor. This complex is required for the activation of *ntcp* transcription [67]. While the expression of FXR was not measured in this study, our findings imply that this mechanism may have been involved, given the significant upregulation of *ntcp* and the downregulation of *shp-2* observed in fish fed the HPM diet compared to those on the FM diet. In the liver, bile acids are synthesized from cholesterol, which serves as their precursor [65]. The cholesterol pool in animal bodies is supplied by dietary intake and by de novo synthesis from acetyl coenzyme A (CoA). Since cholesterol is found only in animal-based products, in the present study, the experimental diets relied solely on fishmeal as their source of cholesterol. Specifically, the HPM diet provided only about 27% of the cholesterol content found in the FM diet (Table 3). Consistently, fish fed the HPM diet demonstrated significantly lower fasting plasma cholesterol levels by the end of the trial (Figure 6A). Furthermore, cholesterol dietary supplementation has been shown to exert positive impacts on Ax bioavailability by promoting Ax absorption [68] and increasing the concentration of Ax in plasma [69] in salmonids, which agrees with our results. Thus, the reduced cholesterol input from the plant-based diet may have impaired bile acid synthesis in the present study. Previous studies have reported similar effects, where carnivorous fish fed plant meal-based diets showed reduced cholesterol concentrations [32,70,71,72,73,74,75,76,77,78] as well as lower bile acid levels [32,78,79] in plasma. On the contrary, Kortner et al. [80] reported that supplementing dietary cholesterol in a plant-based diet completely inhibited cholesterol synthesis and stimulated bile acid production in Atlantic salmon. Overall, these studies suggest a close link between mechanisms regulating cholesterol balance and bile acid homeostasis.

Cholesterol synthesis pathways are well conserved among animals, reflecting their essential role in cellular and metabolic functions [81]. Membrane-bound transcription factors, including those in the SREBP family, are essential for cholesterol synthesis and are involved in feedback mechanisms that ensure cholesterol homeostasis [82]. From these, the SREBP-2 isoform, encoded by *srebp-2*, is most noted for its role in enhancing the expression of genes related to cholesterol biosynthesis [83]. The markedly low cholesterol content of the HPM diet, together with the reduced plasma cholesterol and bile acid levels, indicates that fish fed this treatment experienced a sustained deficit in the cholesterol supply required to sustain bile acid synthesis. In line with canonical sterol homeostasis, the liver responded by upregulating *srebp-2*. A similar effect was reported by Zhu et al. [84], who observed that rainbow trout fed a plant-based diet showed increased *srebp-2* expression; however, in contrast to our findings, those authors detected a reduction in the expression of bile acid synthesis genes such as *cyp7a1*, suggesting that fish may downregulate bile acid production under cholesterol scarcity to minimize sterol efflux. The reasons for this discrepancy remain unclear and warrant further investigation. Evidence from Atlantic salmon further supports our interpretation: when fish were fed a cholesterol-poor plant-based diet, dietary cholesterol supplementation suppressed the entire hepatic cholesterol synthesis pathway, including marked reductions in SREBP-2–regulated genes, whereas the cholesterol-poor condition (i.e., without supplementation) was associated with higher SREBP-2 activity and increased expression of downstream biosynthetic genes [80]. This pattern confirms that low dietary cholesterol reliably triggers SREBP-2–mediated compensatory synthesis in salmonids. However, in our trial, this transcriptional compensation was insufficient to restore circulating bile acid concentrations, revealing a mismatch between regulatory activation and functional output in the cholesterol–bile acid axis. A plausible explanation is that the magnitude of de novo cholesterol synthesis induced by *srebp-2* was simply not sufficient to meet the metabolic demand for bile acid production. Because cholesterol is the rate-limiting precursor for bile acid biosynthesis, a chronic dietary deficit may critically constrain the amount of cholesterol that can be diverted toward *cyp7a1*- and *cyp8b1*-mediated bile acid synthesis, even when their transcription is upregulated. Moreover, we cannot rule out the possibility that, in parallel, bioactive components present in the plant protein blend interfered with the downstream steps of bile acid metabolism or enterohepatic recirculation. Such interference could uncouple the transcriptional response from its functional outcome, particularly since we did not perform targeted analyses to identify or quantify potential antinutritional factors. Under this scenario, the concomitant upregulation of *srebp-2*, *cyp7a1*, and *cyp8b1* in HPM-fed fish would represent an attempt to compensate for low bile acid availability, but this response would remain functionally ineffective, because substrate supply and/or downstream steps of bile acid handling are constrained. This dysregulated compensation provides a coherent mechanistic framework linking chronic cholesterol restriction, impaired bile acid homeostasis, reduced lipid digestibility, and ultimately lower astaxanthin bioavailability in fish fed the high plant protein diet.

For its part, gene expression analysis conducted 6 h postprandially in the distal intestine revealed that the *ostα-2* isoform was the only bile acid transporter whose transcriptional activity was negatively affected by the inclusion of high levels of plant proteins, compared to both the FM and MPM diets at the end of the feeding trial. Remarkably, this agrees with Murashita et al. [37], who detected that the long-term feeding of dietary SMB suppressed the expression of *osta-2* at 6 h postprandial in rainbow trout. The heteromeric organic solute transporter family, comprising OSTα and OSTβ, plays a critical role in maintaining bile acid homeostasis. These transporters work in unison to reabsorb bile acids in the distal intestine and facilitate their return to the liver via the portal circulation. After bile acids are absorbed by enterocytes, the OSTα-OSTβ transport system carries them across the basolateral membrane into the portal bloodstream, ensuring their efficient transport to the liver. OSTα-OSTβ is positively regulated by bile acids via activation of the farnesoid X receptor, a nuclear receptor [85]. Therefore, the downregulation of transporters for bile acids in HPM suggests that this group had low concentrations of bile salts in the digesta (Figure 8). However, bile salts were not measured in the feces in this study, and thus, careful consideration is required when interpreting the results. Finally, FABP-2, encoded by the *fbp-2* gene, is thought to play a key role in fatty acid absorption within the gut. Its expression follows a distinct gradient, progressively decreasing from the proximal to the distal regions of the intestine in fish [56]. Here, we observed that fish fed the HPM diet downregulated the expression of *fbp-2* in the distal intestine compared to those fed either the FM or MPM diets (Figure 8). This suggests a direct link between *fbp-2* expression in the distal intestine and lipid digestibility, given the reduced lipid digestibility in the group consuming the high plant protein blend diet. In line with these results, fish fed the HPM diet exhibited significantly lower lipid retention than those fed either the FM or MPM diets (Table 5), indicating that the HPM-fed group had reduced efficiency in incorporating dietary lipids into fish body tissues. Moreover, the HSI of fish fed the high plant protein blend diet was significantly reduced compared to the other experimental groups. Such effects of high plant protein diets on HSI in carnivorous fish have been documented in earlier studies [1,32,72,74,86]. The histomorphological evaluation of the liver revealed that fish fed the high plant protein blend diet exhibited lower deposition of lipids evidenced by a hepatic tissue with low fat infiltration and hepatocytes in an initial stage of cytoplasmic microvacuoles (Figure 16). Interestingly, similar results were reported by Kortner et al. [32], where higher glycogen and lipid deposition were observed in the hepatocytes of fish fed the fishmeal control diet compared to those fed a soybean meal-based diet. In our study, the hepatic histomorphological findings were further supported by a semi-quantitative scoring system, which revealed higher fat content scores in fish fed the FM diet than in those fed the HPM diet. Consequently, the reduced fat deposition in fish fed the HPM diet likely contributed to lower liver relative weights, reflecting the effects of reduced lipid digestibility caused by the intake of a high plant protein blend diet.

As expected, increasing the inclusion of plant proteins beyond half of the fishmeal level in trout diets, formulated to maintain similar protein, lipid, and energy content, negatively affected growth and productive performance compared to both the fish fed the Fish Meal diet and those fed the Medium Plant Meal diet (Table 5). This effect has long been reported in salmonid fish, and potential factors contributing to this phenomena are well described in previous works [87,88,89,90]. Indeed, replacing fishmeal with plant protein sources often leads to significant reductions in growth performance, protein retention, and PER, with the extent of these effects varying according to the fish species and the type of plant protein used [91]. Lipids are recognized as the principal energy source for fish, playing a crucial role in supporting their metabolic demands and growth performance [92]. In our study, lipid digestibility drops significantly, as fishmeal was increasingly replaced with a plant protein mixture. Given that all experimental diets were formulated to be isonitrogenous, isocaloric, and isolipidic, and that feed intake (relative to body weight) was maintained at a constant across treatments, it can be inferred that the reduced growth observed in fish fed the high plant protein diet was primarily due to impaired lipid digestibility. This, in turn, likely resulted in a diminished availability of metabolizable energy from dietary lipids. Supporting this interpretation, fish consuming the high plant protein diet exhibited significantly lower whole-body lipid content compared to those fed the control diet, indicating reduced lipid deposition as a consequence of decreased lipid utilization.

While it is important to acknowledge the possibility that the high replacement of fishmeal with plant protein may have induced a metabolic disturbance or suboptimal health status in the HPM group—as previously reported in similar studies—we believe this alone does not fully explain the reduced pigmentation observed in this group. The most compelling evidence against the idea that poor pigmentation was merely a secondary consequence of impaired growth lies in the postprandial plasma astaxanthin concentrations, which were significantly lower in fish fed the HPM diet at 24 h post-feeding. This finding strongly supports the notion that the reduced pigmentation in muscle tissue was primarily due to impaired digestibility and absorption of dietary astaxanthin, rather than a downstream effect of general metabolic stress. This specific measurement was incorporated into the experimental design as a physiological marker to disentangle absorption-related effects from other potential confounding factors, such as altered metabolism or growth-related dilution. At the same time, we acknowledge that we cannot entirely rule out additional, more chronic metabolic alterations induced by long-term exposure to plant protein-based diets. Nevertheless, the absorption-related evidence clearly points to limited astaxanthin uptake as a major contributor to the pigmentation differences. Moreover, it is important to highlight that growth performance across treatments remained within acceptable ranges for nutritional trials in fish. In the present trial, fish fed the HPM diet increased from 182 g to 437.5 g over 12 weeks, achieving a 2.4-fold increase in body weight (~240% of initial weight), which is well within the recommended range for fish nutrition trials (≈200–300% over 8–12 weeks), as established by NRC (2011) [93] and Turchini & Hardy (2024) [94]. This supports the conclusion that the fish exhibited adequate growth under the experimental conditions, further reinforcing the role of carotenoid digestibility—rather than general malnutrition—as the primary driver of reduced pigmentation in the HPM group. Therefore, the higher proportion of unhydrolyzed mono- and di-esterified astaxanthin found in the feces of the HPM group (Figure 2B) indicates a reduced capacity to hydrolyze lipid-bound astaxanthin. This observation strengthens the mechanistic link between impaired lipid digestion, alterations in bile acid metabolism, and the consequent reduction in astaxanthin bioavailability. Highlighting this point is essential, as it reinforces the metabolic framework that explains why fish fed the high plant-protein diet showed reduced pigment deposition despite receiving equivalent dietary astaxanthin levels. Finally, when integrated with the observation that replacing approximately 50% of fishmeal with this plant-protein blend did not result in significant differences in growth performance, flesh coloration, muscle astaxanthin concentration, or lipid digestibility, these findings collectively support the existence of a “threshold effect” for fishmeal substitution. In practical terms, partial replacement up to this level appears to preserve key productive and physiological outcomes—such as growth, feed conversion, pigmentation, and nutrient utilization—indicating that this degree of plant-protein inclusion can be achieved without detrimental impacts on salmonid performance.

The analysis of distal intestinal digesta microbiota demonstrated that increasing fishmeal replacement in the diet of rainbow trout did not influence the richness or diversity of the microbiota in digesta samples (Figure 9). It is worth noting that this analysis focused on the digesta (transient) microbiota, distinct from the mucosal (adherent) microbiota, which is closely associated with host metabolism. The impact of replacing fishmeal with alternative feedstuffs on the richness or diversity in gut microbiota of fish varies across studies in the scientific literature. Previous studies have demonstrated that replacing fishmeal with alternative protein sources in fish diets has negligible or no impact on the richness and diversity of gut microbiota [44,95,96,97,98,99,100,101]. In contrast, other research has documented notable alterations in microbial diversity in this feeding context [46,102,103,104,105]. Factors contributing to the inconsistency in the effects of replacing fishmeal with alternative protein sources in fish diets may include the trophic level of the fish, the specific dietary components under evaluation, the variability of test diets, feeding strategies, and the life stage of the fish. The species richness of the microbiota in tank water samples remained similar between the initial sampling and the end of the trial, suggesting that dietary treatments had minimal impact on these aspects of the water microbiota communities during the feeding trial (Figure S4). However, it is important to note that the RAS system used in this study was communal for all tanks rather than individual, which may have masked any potential differences caused by dietary treatments in tank water microbiota species richness. Over the course of the feeding trial, the replacement of fishmeal with the plant meal blend resulted in significant changes to the distal intestinal digesta microbial composition at both medium (MPM) and high (HPM) replacement levels at week 8. These changes were observed when compared to the FM diet group and were further evident when transitioning from MPM to HPM (Table 2). After 12 weeks of feeding, the inclusion of the plant protein meal blend affected microbiota composition only when fishmeal was replaced at the highest level (FM vs. HPM). No dietary effects were observed on the distal intestinal digesta microbial composition at week 4, which can be attributed to differences in composition within groups or heterogenous dispersion (Table 2). Given that all fish shared a common genetic background from the commercial hatchery, the inter-individual variance within groups could suggest an ongoing adaptation process of the gut microbiota to the respective experimental diets, especially in fish receiving the MPM and HPM diets. Interestingly, regardless of the experimental diet, all groups exhibited significant changes in the distal intestinal digesta microbiota composition compared to their initial status on the acclimation diet, highlighting a pronounced effect of the growth process itself on the structure of intestinal microbiota communities (Table S1). This is consistent with previous studies that reported similar results [43,44]. Here, the distal digesta microbiota was dominated by the phyla *Firmicutes*, *Proteobacteria*, and *Actinobacteria* at the start of the study and at both week 4 and week 8, regardless of the experimental diet. This hierarchy order was also observed in the microbiota measured in experimental feed samples and in water tank samples at the end of the study. However, at week 12, the distal digesta microbiota revealed an inversion in the hierarchy in all experimental groups, with *Proteobacteria* becoming the dominant phylum, followed by *Firmicutes* (Figure 10). Similarly, previous studies have documented shifts in gut microbiota composition within the same experimental group over time [43,44,106,107]. Hence, the results of this study indicate that the alterations in the hierarchical structure at the phylum level are more strongly influenced by fish size effects than by diet. The dominance of *Firmicutes* and *Proteobacteria* within the phylum hierarchy of distal intestinal content-associated microbiota has been previously reported in salmonid species both in freshwater [108,109,110,111,112,113] and marine environments [48,109,111]. The gut microbiota is highly sensitive to environmental factors and plays a crucial role in nutrient metabolism and fish health [44]. Replacing fishmeal with plant-based feedstuffs in the diets of carnivorous fish has been reported to influence gut microbial composition. Zhang et al. [114] observed that *Proteobacteria* and *Firmicutes* were the two most abundant phyla in rainbow trout irrespective of the type of diet. According to the authors, fish fed a plant meal-based diet exhibited an increase in *Proteobacteria* and a decrease in *Firmicutes* compared to their counterparts on a fishmeal-based diet. Similarly, Estruch et al. [101] described that feeding a plant meal-based diet to gilthead sea bream (*Sparus aurata*) caused an increase in the relative abundance of *Proteobacteria* and a reduction in *Firmicutes* in distal intestine content. For their part, Desai et al. [43] reported that *Proteobacteria* and *Firmicutes* were the predominant phyla in rainbow trout. However, the study found that fish fed a plant meal-based diet exhibited an increased relative abundance of *Firmicutes* compared to those on a fishmeal-based diet. On the contrary, Gajardo et al. [48] reported that *Firmicutes* was the predominant phylum regardless of the diet and showed a significant increase in relative abundance in the distal digesta compared to the group fed a fishmeal-based diet. Notably, the authors reported that *Proteobacteria* was the predominant phylum in the microbiota associated with the intestinal mucosa, with no changes observed due to the type of diet. Recently, Tawfik et al. [115] reported that *Firmicutes*, *Proteobacteria*, and *Actinobacteriota* were the three most abundant phyla in the distal intestine digesta of Atlantic salmon fed either a fishmeal-based or a plant meal-based diet. In the latter group, the relative abundance of *Firmicutes* was reduced after 6 weeks of feeding. As evidenced, the effect of feeding carnivorous fish a plant meal-based diet on gut microbiota composition at the phylum level is inconsistent across the fish literature, highlighting the challenges in conducting standardized studies that yield reproducible results. Contrary to our expectations, increasing the plant meal blend in the diet did not result in significant changes in the relative abundance of the three major phyla in rainbow trout. Notable differences at the genus level were detected among the experimental diets across all three sampling periods (weeks 4, 8, and 12). Remarkably, the majority of these differences were associated with comparisons between the two most contrasting diets (HPM vs. FM) at each of the evaluated time points (Figure 14A), which indicates that replacing half of the fishmeal in salmonid feeds with plant proteins exerts a marginal effect in microbiota composition.

Finally, Spearman correlation analysis revealed a significant positive association between *Bacillaceae* and astaxanthin (Ax) concentration in muscle and plasma, as well as the digestibility of mono- and di-esterified Ax and filet pigmentation grade, while *Microbacteriaceae* showed a significant negative association with these parameters. Remarkably, Nguyen et al. [39] reported similar findings, observing that *Bacillaceae*, along with other carotenoid-synthesizing bacterial families, was more abundant in Atlantic salmon with darker flesh, while *Microbacteriaceae* was more prevalent in paler fish. *Bacillaceae* is a type of carotenoid-synthesizing bacteria family, and thus, members of this taxa, such as the *Bacillus* species, can synthesize carotenoid pigments. Carotenoid biosynthesis in *Bacillus* species predominantly yields C30 carotenoids, which are structurally distinct from C40 carotenoids, such as astaxanthin. These C30 carotenoids are oxygenated derivatives of apolycopene, the principal carotenoid identified in this bacterial genus [116]. As these bacteria do not synthesize astaxanthin, they cannot serve as direct endogenous sources of this pigment for fish. However, their association with higher astaxanthin levels in trout muscle and plasma suggests a potential interaction with carotenoid metabolism in salmonids, as previously proposed [39]. In the liver, astaxanthin is primarily metabolized by cytochrome P-450 1A1/2 [117], whose activity can be downregulated by retinoic acid receptor/retinoid X receptor (RAR/RXR)-selective ligands [118]. Given their increased aqueous solubility and electrophilicity, C30 carotenoids—such as apolycopene—may more effectively interact with transcription factors like RAR/RXR [119]. However, this remains a speculative interpretation, and further functional studies are necessary to validate this hypothesis to determine whether C30 carotenoids, such as apolycopene, produced by *Bacillus* species and linked to higher astaxanthin levels in fish muscle and plasma, contribute to astaxanthin preservation by reducing its metabolism via cytochrome P-450 1A1/2 at the protein and metabolic levels. Interestingly, the *Shewanellaceae* bacterial family was found to be negatively associated with the inclusion level of plant meal in the diet. This aligns with our previous study, where we observed a significant reduction in the abundance of the *Shewanella* in Atlantic salmon fed either a soybean meal-based or a fermented soybean meal-based diet compared to those fed a fishmeal-based control diet [106]. When integrating these results, a potential mechanistic pathway emerges linking diet composition, gut microbiota structure, and astaxanthin utilization. Higher inclusion of plant proteins reduced the abundance of *Bacillaceae*—a bacterial family that in our study showed positive correlations with lipid digestibility, astaxanthin retention, and plasma Ax levels—and increased families such as Microbacteriaceae, previously associated with paler filets in salmonids. These microbial shifts co-occurred with altered bile acid homeostasis and reduced expression of distal-intestinal lipid transporters, suggesting that microbiota changes may influence, directly or indirectly, bile acid availability and the absorption of lipophilic compounds. However, this interpretation is based solely on correlational patterns and does not establish causation. Therefore, the pathway proposed here should be considered a preliminary mechanistic hypothesis that must be tested in future functional studies (e.g., metagenomics, metabolomics, in vitro assays, or gnotobiotic models) to determine whether these microbial taxa actively contribute to astaxanthin metabolism.

## 4. Materials and Methods

### 4.1. Animal Ethics

Fish management was in line with the recommendations of Guide for the Care and Use of Laboratory Animals of the National Institutes of Health of Chile, and the Committee on the Ethics of Animal Experiments of Universidad Católica de Temuco (Temuco, Región de La Araucanía, Chile), ethic code CEIUCT03280107/25.

### 4.2. Fish and Rearing Conditions

The study was conducted in a freshwater recirculation system at Unidad Experimental de Acuicultura, Universidad Católica de Temuco, Region de la Araucanía, Chile. A total of 495 fish (*O. mykiss*; 131.2 ± 13.5 g) were obtained from a commercial freshwater fish farm (Piscícola Huililco, Pucón, Región de la Araucanía, Chile). Fish were randomly distributed in nine 800 L fiber-glass tanks (55 fish per tank) supplied with freshwater at constant temperature (12 ± 1.5 °C), and aeration was continuously provided via air stones to maintain dissolved oxygen levels near 90 ± 5% oxygen saturation. Fish were acclimated to experimental conditions under a natural photoperiod and fed ad libitum, twice a day, with an acclimatation diet (control diet containing no astaxanthin and no chromium oxide, Cr_2_O_3_) for three weeks until reaching an average body weight of 182 ± 1.5 g. Water quality physiochemical parameters (i.e., oxygen, temperature, pH, and nitrate concentration) were checked on a daily basis.

### 4.3. Diets and Feeding Trial

A blend of plant proteins, mainly soy protein concentrate, wheat gluten, and micronized extruded soybean meal (in a 1:1:1 ratio), was used to replace fishmeal in the MPM and HPM diets at 40% and 80%, respectively, of the fishmeal level (600 g/kg of diet) included in the LPM diet. Three experimental diets were formulated as follows: Low Plant Meal diet (LPM, as the control diet with 0% of the plant protein blend and 60% fishmeal), Medium Plant Meal diet (MPM, with 27% of the plant protein blend and 36% fishmeal), and High Plant Meal diet (HPM, with 54% of the plant protein blend and 12% fishmeal). Experimental diets were formulated to meet the nutrient requirements of *O. mykiss* [93] and to result in similar protein, lipid, and gross energy contents (Table 5). Diets were produced at Centro Tecnológico para la Innovación Alimentaria (CeTA), Santiago, RM, Chile. Ingredients were grinded and thoroughly mixed before the extrusion cooking process with a laboratory twin-screw extruder (Clextral BC-21, Firminy, France). Diets were dried at 80 °C for 1.5 h in a hot air oven until they reached ~6% moisture. Oil was added to experimental diets according to the specific formulations with a vacuum coater Conmetal-2021 (Conmetal, Talcahuano, Región del Bío Bío, Chile). A commercial natural-Ax derived from the microalgae, *Haematococcus pluvialis*, (NatAxtin Oil^®^ (10%); Atacama Bio Natural Products S.A.C., Atacama, Chile) was added as pigment source during the oiling process of diet manufacturing. The product is an oleoresin (60–85%), rich in the (3S,3′S) enantiomer of astaxanthin, extracted from microalgae using supercritical CO2 technology, and mixed with extra virgin olive oil matrix (15–40%). Rapeseed oil was used as cholesterol-free oil source to balance lipid content among experimental diets. Diets were stored at 4 °C, while daily portions were weighted and kept in plastic bags at −20 °C until use. Fish feeding management was conducted similarly to previous studies [18,19,20]. Briefly, each experimental diet was fed to triplicate tanks, and the feeding ratio was fixed at 1.5% (BW day^−1^) of the tank biomass to ensure similar feed intake between experimental groups. Feed was carefully offered by hand, twice a day, during the 12-week feeding trial. Every two weeks, after a 48 h fast, fish in each tank were lightly anesthetized with benzocaine (20%; BZ^®^-20) at a dose of 30 mg/L and individually weighed to adjust the feeding rate during the trial and to monitor growth performance.

### 4.4. Chemical Composition of Diets and Fish, Cholesterol in Diets, and Lipid Quantification in Feces

Chemical analyses of diets and fish were conducted following AOAC methods [120] at Instituto de Nutrición y Tecnología de los Alimentos (INTA), University of Chile. Briefly, dry matter was determined by drying samples overnight (12 h) in an oven at 105 °C to a constant weight. Crude protein content was determined according to Kjeldhal method (the nitrogen-to-protein conversion factor used was 6.25) using a nitrogen analyzer. Crude fat in diets was determined following the Mojonnier method (acid hydrolysis) (AOAC Official Method 935.38, 925.32, 922.06). Ash content was determined by incineration at 600 °C for 4 h. Crude fiber was measured gravimetrically following the Weende method (AOAC Official Method 978.10). Nitrogen-free extract (NFE) was estimated as follows: % NFE = % DM − (% EE + % CP + % ash + % CF). Diet gross energy was determined using a bomb calorimeter (Model C2000 Basic) at Universidad de La Frontera, Temuco, Chile (IKA, Campinas, SP, Brazil). Cholesterol concentration in diets was determined using a Gas Chromatography with FID detector following AOAC Official Method 994.10. The crude fat content in lyophilized feces was determined following the Bligh and Dyer method [121]. Briefly, the collected feces samples were lyophilized (Alpha 1–4 LSC basic, Martin Christ, Osterode, Germany), homogenized, and stored at −80 °C for further analyses. Approximately 1 g of the feces sample were mixed with 5 mL of a chloroform/methanol solution (1:1 *v*/*v*), followed by a mixing and sonication phase of 12 min (repeated 8 times). Then, a separation phase was conducted by adding 2 mL of water to the mixture and 3 mL of chloroform, followed by centrifugation at 10,000 rpm for 20 min. Next, the lipids and chloroform were removed (this step was repeated to ensure all lipids were extracted). Finally, an evaporation and weighing phase was conducted by slowly evaporating the solvent using a temperature digester at 60 °C and weighing the amount of recovered lipids. The chromium oxide content in the diets and feces was estimated according to Furukawa and Tsukahara [122]. All analytical work was performed in triplicate.

### 4.5. Sampling Scheme and Protocols

From the initial fish population, nine fish were sampled to collect distal intestine digesta for microbiota analysis and filet samples to determine pigmentation score and muscle astaxanthin concentration. After a 48 h fast from the acclimatation diet, nine fish were sampled for whole body proximate composition analysis, and nine fish were sampled for the determination of hepatosomatic and viscerosomatic indexes, as well as for hepatic histology to obtain initial values. At the beginning of the 12-week trial (initial time), the blood sampling procedure for determining initial values was conducted as follows: to measure cholesterol concentration and basal levels of total bile acid and astaxanthin concentration in plasma, three fish per tank were sampled after a 24 h fast from the acclimation diet. Next, fish were fed a single morning meal of either experimental diet at half (~0.75%) of the daily feeding ratio (BW day^−1^), with a 15 min gap between tanks to account for blood sampling timing, thus ensuring that sampling occurred at the same postprandial time in each tank. Three fish per tank were sampled 6 h postprandial for total bile acid plasma concentration determination and another three fish per tank were sampled 24 h postprandial for astaxanthin plasma concentration determination. The 24 h postprandial timing for astaxanthin concentration in plasma was set based on previous research indicating that astaxanthin plasma levels reach a maximum between 24 and 42 h postprandial after a single feeding in salmonid species, mainly because of its low intestinal abortion rate [123]. At week 4 and week 8, three fish per tank were sampled to collect distal intestine digesta for microbiota analysis. To obtain final values, at the end of the trail (week 12), three fish per tank were sampled to collect distal intestine digesta for microbiota analysis and filet samples to determine both pigmentation score and muscle astaxanthin concentration. The blood sampling procedure to determine final values of cholesterol and total bile acid was conducted similarly to the initial sampling, with the exception of Ax basal and 24 h postprandially protocol. In order to control for Ax plasma concentration remanent, we proceed as follows: a 72 h wash-out fast period after the last meal was implemented to ensure the clearance of Ax plasma concentration in fish based on previous report [123]. Then, three fish per tank were sampled for blood collection to determine the basal Ax concentration in plasma. This was considered as a covariable to rule out any bypass caused by residual Ax concentration when analyzing the final 24 h postprandial Ax plasma concentration through analysis of covariance (ANCOVA). Next, fish were fed a single morning meal of either experimental diet at half (~0.75%) of the daily feeding ratio; then, after 24 h postprandial, three fish per tank were sampled for blood collection to determine final 24 h postprandial Ax concentration in plasma. Further, two fish per tank were sampled for whole-body proximate composition analysis, and three fish per tank were sampled for the determination of hepatosomatic and viscerosomatic indexes, as well as for hepatic histology. For the collection of samples requiring euthanasia, fish were put to death with an overdose (100 mg/L) of benzocaine (BZ-20^®^; Veterquímica, Santiago, RM, Chile) via an immersive bath to collect whole fish, tissue, and distal intestine digesta samples at each sampling point. Finally, at the end of week 12, the remaining fish were lightly anesthetized with a dose (30 mg/L) of BZ^®^-20 to collect feces to calculate the apparent digestibility of lipids as well as of mono- and di-esterified astaxanthin. Feces were collected by gently stripping the abdomen. Precautions were taken to ensure that the feces did not become contaminated with urine from the fish. Fecal samples were collected in plastic falcon tubes (50 mL), pooled by tank, and immediately frozen until analyses. In addition, samples of water tanks at the beginning and at the end of the trial as well as diet sample were collected for microbiota analyses. The trial experimental design and sampling scheme is illustrated in Figure S1. The 6 h postprandial sampling point for genes expression was selected, since it is a well-established physiological window in salmonids for capturing peak postprandial metabolic and transcriptional responses [124,125,126].

### 4.6. Blood Sample Processing

Blood samples were taken from the caudal vein of slightly anesthetized fish using a syringe; blood was gently transfer to precooled ethylenediaminetetraacetic acid (EDTA) vacutainer tube. Immediately after blood collection, samples were centrifuged at 4000× *g* for 5 min at 4 °C, and plasma aliquots were stored at −80 °C, pending determination of cholesterol, total bile acids, and astaxanthin concentration.

### 4.7. Analyses of Astaxanthin in Pigment Source, Diet, Feces, Muscle, and Plasma

The analysis of astaxanthin in pigment source, diet, and feces were performed at Núcleo Científico Tecnológico en Biorecursos, Universidad de La Frontera (BIOREN-UFRO) (https://bioren.ufro.cl). The concentration of mono and di-esterified astaxanthin as well as free astaxanthin was determined in the pigment source (NatAxtin Oil (10%), diets, and feces following the protocol developed by Todorović et al. [127], using HPLC-DAD (Shimadzu, Kyoto, Japan) analytical mode, Poroshell 120 EC-C18 column (Agilent Technologies, Santa Clara, CA, USA), mobile phase A: H_2_O:MeOH and mobile phase B: MeOH:MTBE, and flow rate of 0.2 mL/min in gradient mode for 55 min. The wavelength for the identification and quantification of astaxanthins is 475 nm. The retention time for free astaxanthin is 8.2 min, for mono-esterified astaxanthin it is 24.4 min, and for di-esterified astaxanthin it is 35.3 min. Each compound is quantified using the equation of the free Ax curve. The results were expressed in milligrams of astaxanthin per gram of raw material and the calibration curve for free astaxanthin. The limit of detection (LOD) was 0.03 μg/mL, the limit of quantification (LOQ) was 0.11 μg/mL, and the linear range was 0–30 μg/mL. The concentration of astaxanthin in flesh muscle and plasma were performed at the certified laboratory of Mérieux NutriSciences, Puerto Montt, Chile (https://www.merieuxnutrisciences.com/cl/). Briefly, samples were homogenized, and an aliquot was weighed (~0.5 g). Water was removed from each sample using a desiccant with hygroscopic properties. The extraction of astaxanthin was performed with an organic solvent using a tissue homogenizer. The astaxanthin concentrations of muscle or plasma samples were calculated using the peak areas from the chromatograms. The samples were injected into an HPLC system in reverse phase using a UV/Visible detector at 470 nm, employing a C18 HPLC Column 5 μm particle size, pore size 100 Å, L × I.D. 150 mm × 4.6 mm, and using Methanol–Acetone as the mobile phase. The concentration was calculated through an external calibration curve using an external standard of astaxanthin (Sigma Aldrich, St. Louis, MO, USA) with a known concentration in triplicates [128].

### 4.8. Calculations of Apparent Digestibility Coefficients (ADCs) and Muscle Retention of Astaxanthin

The ADCs for lipids and mono and di-esterified astaxanthin were determined based on the following formula [129]:

ADC (%) = [1 − (Cr_2_O_3_ in feces/Cr_2_O_3_ in diet) × (nutrient in feces/nutrient in diet)] × 100

Retention of astaxanthin in fish muscle was computed as the ratio of astaxanthin accumulated in the muscle, relative to astaxanthin intake, and expressed similar to previous reports [55,130]:

Ax muscle retention (%) = 100% × (*W*_mu_*/W*_f_) × [(*W*_f_ × Ax_f_) − (*W*_i_ × Ax*_i_*)]/[(*W*_f_ − *W*_i_) × FCR × Ax_feed_]

where Ax_i_ and Ax_f_ are the initial and final astaxanthin concentrations in the muscle, respectively, FCR is the feed conversion ratio, and *W*_i_ and *W*_f_ represent initial and final weights of the fish, respectively. Ax_feed_ is the astaxanthin concentration in the feed (mg kg^−1^). The *W*_mu_*/W*_f_ ratio represents the proportion of white muscle mass relative to the whole-body weight of the fish, which has been reported to be close to 0.6 in rainbow trout [86,131,132,133].

### 4.9. Filet Color Analysis

The color of the filets was determined in two points (loin and belly) using the SalmoFan^TM^ (DSM-Firmenich, Netherlands). The analysis was performed at Mérieux NutriSciences (Puerto Montt, Chile) via a Vision System for Color Classification and Irregularity Detection in salmon filets developed by Lythium SpA [134]. This system has been already validated and implemented to evaluate the quality of the salmon filet in real time, based in processing plants and farming centers in Chile [70]. Briefly, a monitoring station that integrates a camera, a laboratory scanner, and a lighting system, all connected to a computer system, collects and processes the photographed samples using machine learning and image processing. The system captures an image using a camera to process and segments the data that needs interpretation or analysis, dividing the image into areas. The techniques used for this stage include thresholding, discontinuities, region growing, use of color or movement, etc. Once the image is divided, feature extraction takes place, in this case, focusing on the color of the flesh. This way, the data is transformed into more elaborate visual information to objectively measure the color of the salmon filet according to the DSM SalmoFan scale, as well as to detect real-time irregularities such as melanosis, bruising, gaping, and cracking. After the image is segmented and the desired features are extracted, a series of evaluations are performed on these features to determine imperfections.

### 4.10. Analysis of Total Cholesterol and Total Bile Acids in Plasma

The analysis was performed at Núcleo Científico Tecnológico en Biorecursos, Universidad de La Frontera (BIOREN-UFRO) (https://bioren.ufro.cl). Total cholesterol in plasma was analyzed using the cholesterol liquicolor kit (Human Gesellschaft für Biochemica und Diagnostica mbH, Wiesbaden, Germany) following manufacture’s recommendations. Briefly, 10 µL of plasma sample with 1000 µL of working reagent was incubated for 10 min at 25 °C and read at 500 nm using a multimodal Synergy HT reader (BIOTEK, Winooski, VT, USA). For total bile acid determination, aliquots of plasma samples were diluted 1:8 in deionized water. Samples were quantified according to the manufacturer’s protocol (CELL BIOLABS, INC, San Diego, CA, USA); 50 uL of paired samples, diluted and standard, were added to the microplates, and 50 uL of assay reagent was added to the standard well and to a paired sample. Then, 50 uL of NAD^+^ reagent was added to the other half of the paired sample wells. Subsequently, 100 uL of buffer was added to all wells, mixed, and shaken. The microplates were incubated for 60 min at 37 °C in the dark. Fluorescence intensity λ exc 560 nm/λ em 590 nm was measured on a Synergy HT multimodal reader (BIOTEK, Winooski, VT, USA). All the analyses were conducted in triplicate.

### 4.11. Gene Expression Analysis

Tissues samples from liver, midgut, and hindgut were processed for total RNA isolation using TRizol according to the manufacturer’s recommendations (Invitrogen, Rockville, MD, USA). Total RNA concentration was quantified spectrophotometrically using a Qubit 3.0 fluorometer (Thermo Fisher Scientific, Waltham, MA, USA). Aliquots with 5 mg of RNA were reverse transcribed using Maxima H Minus First Strand cDNA Synthesis Kit with dsDNase and oligodT as primer, following the manufacturer’s protocol (Thermo Fisher Scientific, Waltham, MA, USA). Primer sequences for genes of interest and reference genes were either obtained from previous publications or identified using sequences for *O. mykiss* available in the GenBank database (NCBI) (Table 6). They were then designed and analyzed using the PrimerQuest and OligoAnalyzer tools available on the Integrated DNA Technologies website (https://www.idtdna.com). The genes of interest evaluated in liver included, cholesterol 7α-hydroxylase (*cyp7a1*), sterol 12α-hydroxylase (*cyp8b1*), small heterodimer partner (*shp*), bile salt export pump (*bsep*), Na+-taurocholate cotransport peptide (*ntcp*), ATP binding cassette transporter (*abcg8*), and sterol regulatory element binding protein (*srebp-2*). The genes of interest evaluated in hindgut included apical sodium-dependent bile salt transporter (*asbt*), heteromeric organic solute transporter (*ostα*), and fatty acid binding protein 2 (*fabp2*). Gene expression was determined by RT-qPCR using an AriaMX Real-Time PCR System (Agilent, Santa Clara, CA, USA) and the LightCycler 480 SYBR Green I Master kit (Roche, Indianapolis, IN, USA). cDNA was loaded at a concentration of 2 ng in a final 10 µL PCR reaction. Nuclease-free water was used as a negative control, and each reaction was performed in triplicate. Amplification efficiency for each gene was assessed using a standard curve with five different concentration points. The PCR cycling conditions included an initial denaturation at 95 °C for 10 min, followed by 45 cycles of 95 °C for 5 s, 65 °C for 10 s, and 72 °C for 10 s. Specific amplifications for each gene were sequenced to confirm their identity. In each assay, the melting curve was analyzed to identify any nonspecific amplifications. Gene expression data were analyzed using the 2^−ΔΔCt^ method as described by Livak and Schmittgen [71]. The expression levels of the target genes were normalized against the geometric mean of elongation factor 1-alpha 1 (*elf-1a*) and beta actin (*β-actin*). The normalized gene expression of fish fed either the MPM or the HPM diet was divided by the normalized gene expression of the control group (FM diet).

### 4.12. DNA Extraction and Sequencing

DNA extraction procedure was similar to previous study [44]. Briefly, DNA was extracted from homogenized lysates of distal intestinal digesta (approximately 0.25 g) using the MOBIO PowerFecal DNA Isolation Kit (MO BIO Laboratories, Carlsbad, CA, USA), following the manufacturer’s instructions. DNA concentrations were determined using the Qubit dsDNA HS Assay Kit (Life Technologies, Grand Island, NY, USA). The V4 region of the 16S rRNA gene was amplified using the fusion primer method with the primers 515F (5′-GTGCCAGCMGCCGCGGTAA-3′) and 806 R (5′-GGACTACHVGGGTWTCTAAT-3′). The resulting amplicons were of appropriate length for sequencing on the Illumina platform (San Diego, CA, USA). All PCR reactions were conducted in duplicate in a 30 µL reaction mixture containing 1.5 U (5 U/mL) of GoTaq G2 Flexi DNA polymerase (Promega, Madison, WI, USA), 6 µL of 5× buffer, 2.4 µL of 25 mmol/L MgCl_2_, 1.2 µL of a 5 mmol/L nucleotide mix, 0.3 µL of 20 mmol/L primers, and 18.5 µL of nuclease-free water. A negative PCR control without a DNA template was also included. The PCR conditions consisted of initial denaturation at 94 °C for 5 min, followed by 35 cycles of denaturation at 94 °C for 30 s, annealing at 56 °C for 30 s, and extension at 68 °C for 45 s. After cycling, the amplicons from each sample were pooled and run on a 1% agarose gel. The amplicons were then purified using the QIAquick PCR Purification Kit (Qiagen, Germantown, MD, USA). Libraries were sequenced on the Illumina HiSeq PE250 platform configured for 300 bp paired-end reads at CD Genomics (http://www.cd-genomics.com).

### 4.13. Bioinformatics Analysis of Microbial Communities

The raw demultiplexed sequence reads were first evaluated in FastQC software Version 0.12.0 [136], and then the adapter and primer sequences were removed using the cutadapt algorithm [137]. The reads filtered in the previous step were imported into the R environment version 4.4.0 [138]. First, using the *DADA2* library [139], forward and reverse reads were truncated at 225 and 215 bp, respectively, and low-quality reads were removed using the filterAndTrim function, with standard parameters set based on read quality scores for the dataset (maxN = 0, truncQ > 2, and maxEE = 2). Paired reads were assembled after error modeling and correction, creating amplicon sequence variants (ASVs). Then, chimeric sequences were removed using the remove BimeraDenovo function. The ASVs resulting were assigned taxonomy by classifying them in the Silva v138.1 database using a naïve Bayesian classifier, with a minimum bootstrap confidence of 80%. The count table, taxonomy assignment results, and metadata table were compiled into a phyloseq object using the *phyloseq* library [140]. Alpha diversity was assessed using Chao1 and Shannon indices. Singleton ASVs, those assigned as *Cyanobacteria*, *Chloroflexi*, as well as those not assigned at phylum level, were removed. The *ggplot2* library [141] was used to generate the plots.

### 4.14. Histological Analysis

Samples from liver were dehydrated using a graded series of alcohols (from 70% to 100%), cleared in xylene, and embedded in paraffin. Paraffin sections 5 µm thick were stained with hematoxylin-eosin to evaluate cell morphology and fat droplets-derived cytoplasmic vacuoles. Grade of steatosis was determined using a semiquantitative scoring method, as previously reported [44]. The grade of steatosis is determined based on the proportion of hepatocytes containing visible macrovacuoles (fat droplets) and is expressed semi-quantitatively on a scale from 0 to 4: 0 (<5%), 1 (5% to 25%), 2 (25% to 50%), 3 (50% to 75%), and 4 (>75%). All histological samples were analyzed under an Olympus CX21 light microscope (Olympus, Center Valley, PA, USA). The analyses were performed at Laboratorio de Morfología, Programa de Anatomía y Biología del Desarrollo, Facultad de Medicina, Universidad de Chile.

### 4.15. Statistical Analysis

Statistical analyses and graphs were obtained using GraphPad Prism version 10.4 for Mac (GraphPad Software, Boston, MA, USA). Data were analyzed for normality (Kolmogorov–Smirnov test) and homoscedasticity (Levene’s test). One-way analysis of variance (ANOVA) was performed to detect significant effect of using the plant meal blend as fishmeal replacement. Tukey’s HSD tests were performed as post hoc tests to identify experimental groups that differed significantly. In those cases, where either the normality assumption or the homoscedasticity assumption was violated, the Kruskal–Wallis rank sum test was run to determine significant differences between experimental groups, followed by Dunn’s multiple comparisons test as a post hoc test to identify which experimental groups differed significantly. For the final Ax plasma concentration at 24 h postprandial, an analysis of covariance (ANCOVA) was conducted, considering basal Ax plasma concentration (after a 72 h fasting period) as a covariate. In cases where the homoscedasticity assumption was violated for gene expression data, the data were transformed using the natural logarithm (ln). For microbiota analysis, Shapiro–Wilk test was used for normal distribution analysis. A two-way ANOVA (time and diet as factors) with Tukey’s HSD multiple range test was used to evaluate differences in alpha diversity indices. The Aitchison distance was calculated as a metric of beta diversity. First, the transform function of the microbiome library [142] was used for the centered log transformation (clr) of the count table, and then, using the vegan library [143], the Euclidean distance was calculated. Significant differences between the bacterial communities from different groups were evaluated using PERMANOVA. Additionally, the influence of dispersion among groups was determined through Betadisper analysis (permutation test for homogeneity of multivariate dispersions). ASV-level counts were agglomerated by each taxonomic level, and bias-corrected microbiota composition analysis 2 (ANCOM-BC2) was used to test differential abundance. Taxa were defined as significantly differentially abundant in each condition if their *p* value was less than 0.05. The R ANCOM-BC2 library [144] was used to deal with zero inflation and overdispersion in the count data. Finally, Spearman’s correlation analysis was used to assess the correlation between taxa and variables evaluated using the corrplot library after determining that the data were non-normal (Shapiro test, *p* < 0.05) [145]. For this, bacterial genera present in at least 4 out of 9 replicates per diet and with an average relative abundance of 1% in at least one group/diet during week 12 were selected for correlation analysis. First, the ASV table with absolute abundance data was used, and a CLR (centered log-ratio) transformation was applied to ASV counts. Subsequently, variables measured in the fish were included.

## 5. Conclusions

The progressive replacement of fishmeal with plant protein blends in rainbow trout diets demonstrated threshold-dependent effects on muscle pigmentation and astaxanthin (Ax) deposition. Substitution of up to 50% fishmeal (36% inclusion) preserved filet pigmentation and Ax concentrations, whereas higher plant protein inclusion (12% fishmeal) adversely impacted both parameters. This decline correlated with compromised lipid digestibility and reduced Ax retention efficiency, likely driven by diminished dietary cholesterol availability and subsequent alterations in bile acid metabolism. These factors impaired the absorption of liposoluble compounds, particularly di-esterified Ax, which is dependent on efficient micellar solubilization mediated by bile acids. The observed modulation of gut microbiota composition, including reduced abundance of *Bacillaceae*—a bacterial family positively associated with Ax metabolism—further suggests microbial involvement in carotenoid utilization. These findings underscore the importance of balancing plant protein inclusion with cholesterol and bile acid homeostasis to optimize Ax assimilation in salmonids, since it is a critically important antioxidant carotenoid for fish health and filet quality.

## Figures and Tables

**Table 1:** Fish productive variables and biometrics of experimental groups fed either experimental diet for 12 weeks.

	Experimental Diets						Statistical Test
	FM		MPM		HPM		
Productive Variables	Mean	SEM	Mean	SEM	Mean	SEM	*p* Value
Initial weight, g/fish ^1^	182.6	2.1	184.2	1.3	182.1	1.2	0.65 ^€^
Final weight, g/fish ^1^	554.8 ^a^	3.3	520.7 ^a^	18.7	437.5 ^b^	13.6	0.002 ^€^
Relative weight gain, %/fish ^1,4^	203.9 ^a^	1.8	182.7 ^a^	10.9	140.2 ^b^	8.3	0.003 ^€^
FCR ^1,5^	1.1 ^b^	0.0	1.1 ^b^	0.1	1.4 ^a^	0.1	0.02 ^€^
SGR ^1,6^	1.3 ^a^	0.0	1.2 ^a^	0.0	1.0 ^b^	0.0	0.003 ^€^
Condition factor ^1,7^	1.4	0.0	1.4	0.0	1.4	0.0	0.92 ^€^
Protein retention (%) ^2,8^	34.6 ^a^	0.8	29.3 ^ab^	1.8	25.0 ^b^	1.1	0.004 ^€^
Lipid retention (%) ^2,8^	50.7 ^a^	5.0	46.1 ^a^	7.4	22.1 ^b^	0.8	0.02 ^€^
PER ^9^	1.9 ^a^	0.0	1.5 ^b^	0.1	1.2 ^b^	0.1	0.001 ^€^
Initial HSI (%) ^3,†^	1.7	0.2	1.8	0.2	1.7	0.1	0.87 ^€^
Final HSI (%) ^3,†^	1.6 ^a^	0.2	1.5 ^ab^	0.2	1.1 ^b^	0.0	0.01 ^Š^
Initial VSI (%) ^3,‡^	8.5	1.1	7.6	0.8	8.0	0.3	0.76 ^€^
Final VSI (%) ^3,‡^	8.6	1.1	7.7	0.4	6.7	0.4	0.13 ^Š^

^1^ Mean values with their SEM based on bulk tank; three tanks per group (*n* = 3). Different letters indicate significant difference between treatments in the same row (*p* < 0.05). ^2^ Mean values with their SEM for two fish per tank; three tanks per group (*n* = 6). Different letters indicate significant difference between treatments in the same row (*p* < 0.05). ^3^ Mean values with their SEM for three fish per tank; three tanks per group (*n* = 9). Different letters indicate significant difference between treatments in the same row (*p* < 0.05). ^4^ Relative weight gain was estimated as [(g mean final weight − g mean initial weight)/g mean initial weight] × 100. ^5^ Feed conversion ratio (FCR) was calculated as (feed intake/wet weight gain). ^6^ Specific growth rate (SGR) was determined as (ln final weight − ln initial weight/number of trial days) × 100. ^7^ Condition factor was computed as 100 × final body weight/body lenght^3^. ^8^ Nutrient retention was computed as 100 × [(final body weight × final body nutrient content) − (initial body weight × initial body nutrient content))/nutrient intake]. ^9^ Protein efficiency ratio (PER) was determined as weight gain (g)/nutrient intake (g, DM). ^†^ Hepatosomatic index (HIS) was estimated as (100 × (liver weight/body weight)). ^‡^ Viscerosomatic index (VSI) was estimated as (100 × (viscera weight/body weight)). ^€^ One-way ANOVA; Tukey multiple comparisons test. ^Š^ Kruskal–Wallis test; Dunn’s multiple comparisons test.

**Table 2:** Chemical composition of the whole body of rainbow trout fed the experimental diets for 12 weeks ^1^.

			Experimental Diets						Statistical Test ^†^
	Initial Values		FM		MPM		HPM		
Chemical Composition (%)	Mean	SEM	Mean	SEM	Mean	SEM	Mean	SEM	*p* Value
Dry matter	26.9	0.3	31.5 ^a^	0.5	31.8 ^a^	1.0	28.7 ^b^	0.5	<0.05
Crude protein	15.6	0.1	17.5 ^b^	0.2	18.0 ^ab^	0.5	18.3 ^a^	0.1	<0.05
Crude fat	8.1	0.2	12.1 ^a^	0.6	11.7 ^a^	1.0	8.3 ^b^	0.5	<0.05
Ash	2.8	0.1	1.3	0.05	1.5	0.1	1.5	0.1	0.29

^1^ Mean values with their SEM for two fish per tank; three tanks per group (*n* = 6). Different letters indicate significant difference between treatments in the same row (*p* < 0.05). ^†^ One-way ANOVA; Tukey’s multiple comparisons test.

**Table 3:** Astaxanthin concentration in pigment source and experimental diets ^1^.

	Pigment Source		Experimental Diet					
			FM		MPM		HPM	
Astaxanthin (mg/kg)	Mean	SEM	Mean	SEM	Mean	SEM	Mean	SEM
Mono-esterified	113.6	0.0	53.3	2.9	57.8	3.2	54.5	1.4
Di-esterified	13.7	0.0	2.3	0.2	3.2	0.3	2.8	0.3
Free	3.4	0.0	1.5	0.0	1.6	0.0	1.6	0.0
Total ^2^	130.7	-	57.1	-	62.6	-	58.9	-

^1^ Mean values with their SEM analyzed in triplicates. ^2^ Determined as the sum of mono and di-esterified astaxanthin and free astaxanthin.

**Table 4:** PERMANOVA and Betadisper results in digesta samples using Aitchison distance, analyzing the effect of diet on the dataset separated by weeks ^1,2^.

	PERMANOVA		Betadisper
Pairwise Comparison	R^2^	*p* Value	*p* Value
Diet—4 wk	0.104	0.020	0.001
FM vs. MPM	0.054	0.758	0.302
FM vs. HPM	0.086	0.013	0.001
MPM vs. HPM	0.092	0.019	0.006
Diet—8 wk	0.119	0.001 *	0.358
FM vs. MPM	0.088	0.005 *	0.383
FM vs. HPM	0.112	0.001 *	0.210
MPM vs. HPM	0.074	0.048 *	0.531
Diet—12 wk	0.085	0.280	0.670
FM vs. MPM	0.066	0.316	0.684
FM vs. HPM	0.081	0.006 *	0.424
MPM vs. HPM	0.045	0.943	0.608

^1^ Within a row, asterisk (*) indicates significant difference between treatments (*p* < 0.05). ^2^ Experimental diets: Fish Meal diet (FM), Medium Plant Meal diet (MPM), and High Plant Meal diet (HPM).

**Table 5:** Ingredients and nutrient composition of the experimental diets fed to rainbow trout for 12 weeks.

Ingredients (%)	Diets		
	FM Diet	MPM Diet	HPM Diet
Super prime fishmeal ^1^	60.0	36.0	12.0
Soy protein concentrate ^2^	0.0	9.0	18.0
Wheat gluten ^2^	0.0	9.0	18.0
Extruded micronized soybean meal ^3^	0.0	9.0	18.0
Wheat meal ^4^	15.0	12.0	7.5
Alpha cellulose ^2^	4.0	2.0	0.0
Rapeseed oil ^5^	18.0	19.0	20.0
Vitamin premix (including vitamin C) ^3,†^	1.6	1.6	1.6
Mineral premix ^3,‡^	0.15	0.15	0.15
Choline chloride (70%) ^3^	0.57	0.57	0.57
Dicalcium phosphate ^3^	0.0	0.6	2.5
L-Methionine ^3^	0.0	0.1	0.4
Lysine ^3^	0.0	0.3	0.6
NatAxtin Oil 10%^®^ (10% of astaxanthin) ^6^	0.08	0.08	0.08
Chromium oxide (Cr_2_O_3_) ^7^	0.6	0.6	0.6
Feed nutrient composition (as-is, %)			
Dry matter	94.6	95.2	94.0
Crude protein	46.3	47.7	47.7
Fat	22.6	22.4	22.3
Ash	10.0	8.2	7.4
Fiber	1.4	1.1	1.0
Nitrogen-Free Extract (NFE) Gross energy (MJ kg^−1^)	16.3 22.5	15.8 22.9	15.6 22.7
Cholesterol (g kg^−1^)	1.5	1.1	0.4

^1^ Lota Protein, Concepción, VIII Región, Chile. ^2^ Oregon Chem Group, Santiago, RM, Chile. ^3^ QualityPro, Santiago, RM, Chile. ^4^ Molino La Estampa, Santiago, RM, Chile. ^5^ Kirkland Signature, Costco Wholesale Corporation, Issaquah, Washington, USA. ^6^ NatAxtin Oil (10%)^®^, Atacama Bio Natural Products S.A.C., Atacama, Chile (https://www.nataxtin.cl). ^7^ Merck KGaA, Darmstadt, Germany. ^†^ Per kg dry diet: vitamin A 24,000 IU; vitamin D 11,200 IU; vitamin E 240 IU; vitamin C 4.8 g; thiamine 15 mg; riboflavin 30 mg; nicotinic acid 25 mg; pyridoxine 15 mg; cyanocobalamine 0.05 mg; pantothenic acid 50 mg; biotin 0.5 mg; and folic acid 1.58 mg. ^‡^ Contributed in mg kg^−1^ of diet: iron 52.5 mg; potassium 3 mg; manganese 75 mg; zinc 154.5 mg; copper 3 mg; iodine 0.6 mg: cobalto 0.3 mg; and selenium 0.2 mg.

**Table 6:** Primer sequences for *Oncorhynchus mykiss* used in real-time polymerase chain reaction.

Gene	Primer Sequence (5′–3′)	Publication
Cholesterol 7α-hydroxylase (cyp7a1-2) ^1^	F: ACAGGCCAACACACTGCCTGCTACT R: CCGGGAGAGAGTGAGTTGTGGTTTGCT	[37]
Sterol 12α-hydroxylase (cyp8b1-1) ^1^	F: CACAGTGTAGGGACAAAGCATGATAGAA R: CGGGGATTTGGGTGTCTCGTTAC	[37]
Sterol 12α-hydroxylase (cyp8b1-2) ^1^	F: GTGTAGGGACGGGGGATAATAACC R: GGCTTTCTCCATGCTTTCTGTGGA	[37]
Small heterodimer partner (shp-1) ^1^	F: GGAGCTATGCTGTTCAATCCAGACA R: GTAAGTCAGAGGTCGATAGTAGGATGCA	[37]
Small heterodimer partner (shp-2) ^1^	F: GGAGCTATGCTGTTCAATCCAGACA R: GTAAGTCAGAGGTCGATAGTAGGATGCA	[37]
Bile salt export pump (bsep) ^1^	F: CGGCTTCGCCCAGTGTGTCG R: CCCAGCGCTGTGCCACTGGT	[37]
Na+-taurocholate cotransport peptide (ntcp) ^1^	F: CTCTCAGATCATCATCAAGGTTGGTC R: GTGAGAGAACCACCCACACTGTTCC	[37]
ATP binding cassette transporter (abcg8) ^1^	F: GATACCAGGGTTCCAGAGCA R: CCAGAAACAGAGGGACCAGA	[37]
Sterol regulatory element binding protein 2 (srebp-2) ^1^	F: TAGGCCCCAAAGGGATAAAG R: TCAGACACGACGAGCACAA	[37]
Apical sodium-dependent bile salt transporter (asbt) ^2^	R: TGGCTGGATGGAGACATGGACCTCAGT F: TGGATGGTGTCAGCAGAGGTCCAGACAG	[37]
Heteromeric organic solute transporter (osta-1) ^2^	R: AACATCACACGACGGAGTCTGTTCC F: CTCCCGTTATCGCCATATCTGCGAT	[37]
Heteromeric organic solute transporter (osta-2) ^2^	R: CCATGTTAACATCACACGACGGAGTT F: CTCCTGTTACCGCCGTATCTGTAAC	[37]
Fatty acid binding protein 2 (fabp2) ^2^	R: GTACCTGGGAGATGGATGGA F: GCATCCACCCCATCGTAGTT	[69]
Beta actin (b-actin) ^1,2^	R: TACAACGAGCTGAGGGTGGC F: GGCAGGGGTGTTGAAGGTCT	[135]
Elongation factor -1 (elf-1) ^1,2^	R: TCCTCTTGGTCGTTTCGCTG F: ACCCGAGGGACATCCTGTG	[79]

^1^ Analyzed in liver. ^2^ Analyzed in hindgut.

## Data Availability

The data presented in this study are openly available in [NCBI] [https://www.ncbi.nlm.nih.gov/search/all/?term=PRJNA1219833], accessed on 30 November 2025.

## Supplementary Materials

The following supporting information can be downloaded at: https://www.mdpi.com/article/10.3390/ijms262412072/s1.
