# Supplementary material for: High Dietary Plant Protein Impairs Astaxanthin Pigmentation in Rainbow Trout by Disrupting Cholesterol–Bile Acid Metabolism and Gut Microbiota
Source: Int J Mol Sci. 2025 Dec 15;26(24):12072. doi: 10.3390/ijms262412072 (PMC12732709; doi:10.3390/ijms262412072)
Supplement: Supplementary file 1 [file ijms-26-12072-s001.zip › Supplementary material Ax Figs.pdf]

SUPPLEMENTARY MATERIAL

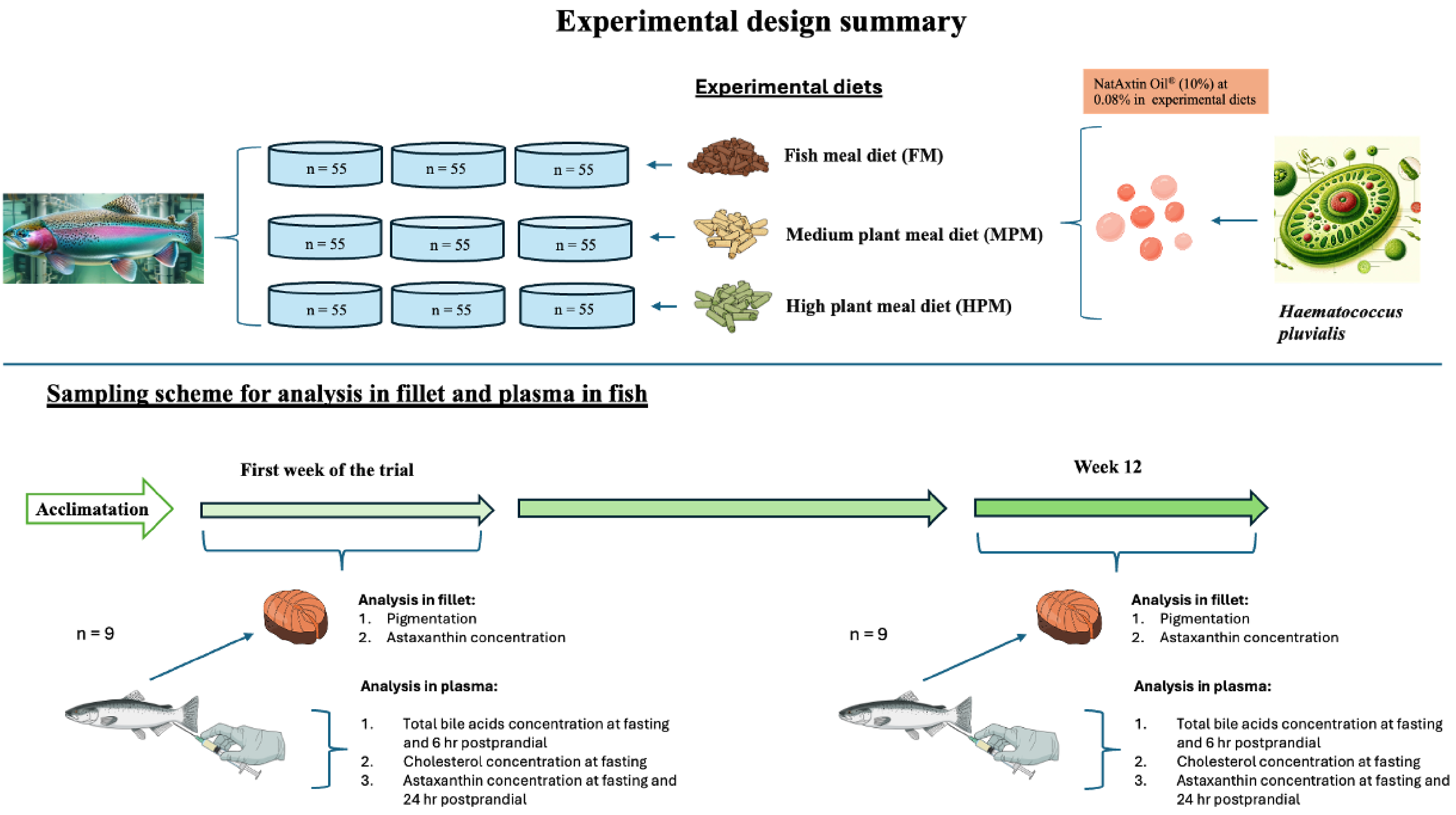

Figure S1. Summary of experimental design and sampling scheme.

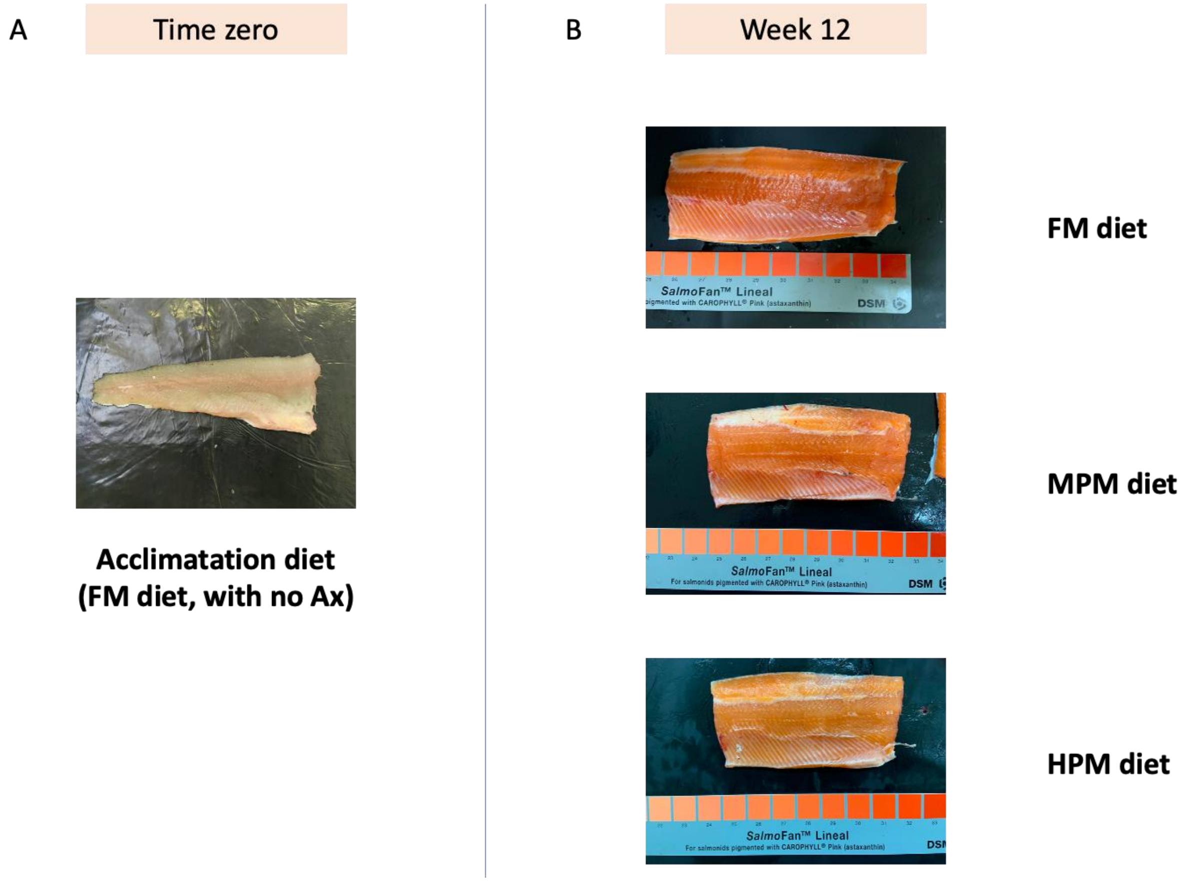

Figure S2. Fillet pigmentation of rainbow trout A) fed the acclimatation diet at the beginning (Time zero) of the feeding trial and B) fed experimental diets at the end (week 12) of the feeding trial. Experimental diets: Fish Meal diet (FM), Medium Plant Meal diet (MPM), and High Plant Meal diet (HPM).

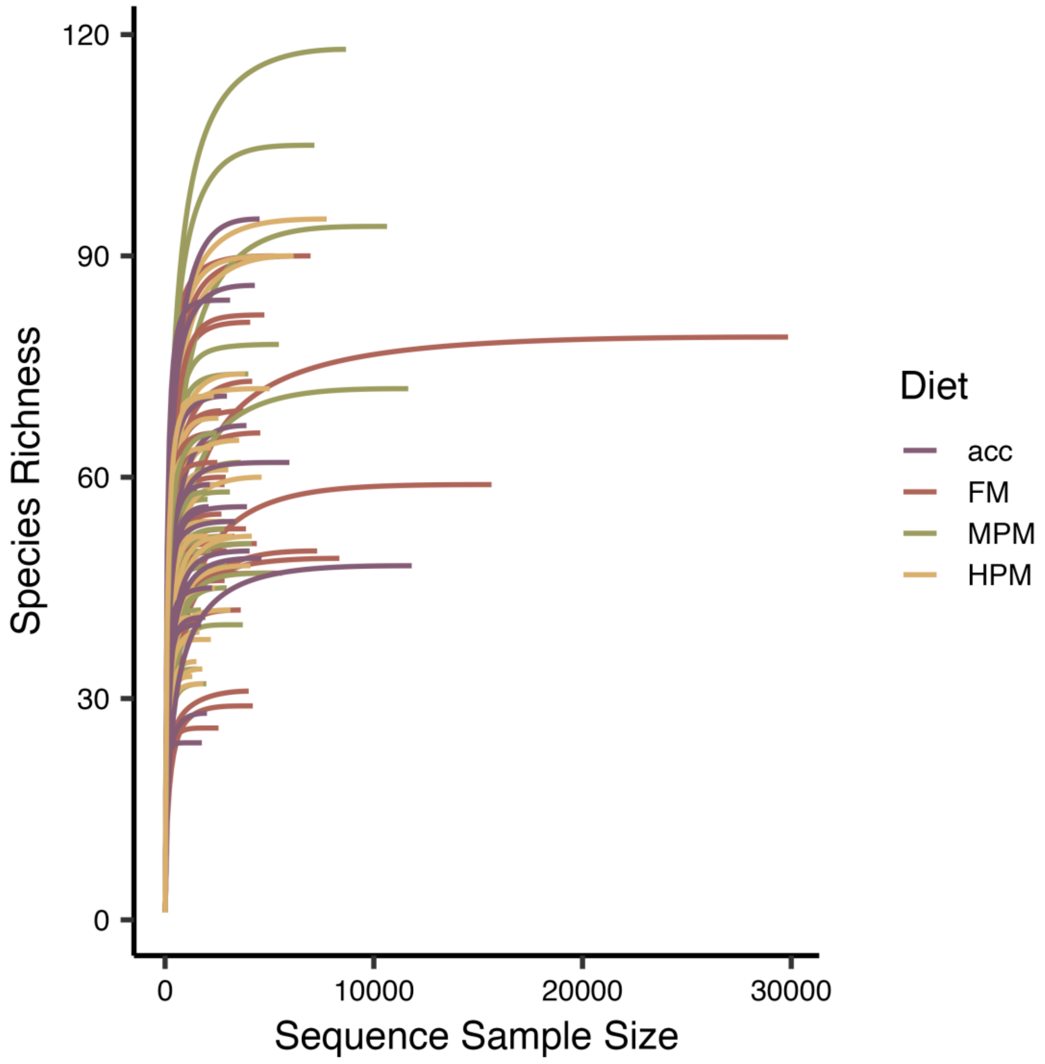

Figure S3. Rarefaction curves based on the richness index.

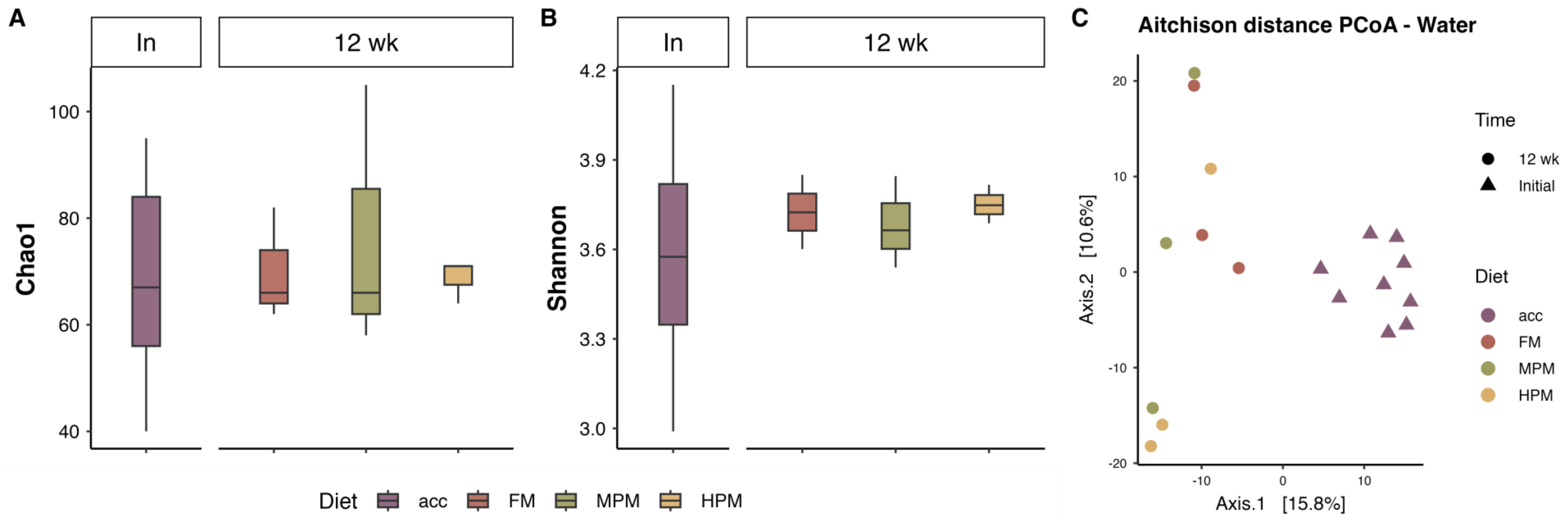

Figure S4. (A) Species richness and (B) diversity of the microbiota found in the water samples at the beginning and at the end of the 12- week feeding trial.
